# Time-Resolved Analysis of Protein–Protein Ensembles Using a Destabilizing Domain to Map Dynamic Interactions of SARS-CoV‑2 nsp15

**DOI:** 10.1021/acschembio.5c00377

**Published:** 2025-09-01

**Authors:** Crissey Cameron, R. Mason Clark, Adam M. Metts, Runze M. Jiang, Toya D. Scaggs, Kwangho Kim, Gary A. Sulikowski, Lars Plate

**Affiliations:** † Department of Chemistry, 5718 Vanderbilt University, Nashville, Tennessee 37235, United States; ‡ Department of Biological Sciences, Vanderbilt University, Nashville, Tennessee 37235, United States; § Vanderbilt Institute of Chemical Biology, Molecular Design and Synthesis Center, Vanderbilt University, Nashville, Tennessee 37235, United States; ∥ Department of Pathology, Microbiology and Immunology, Medical Center, Vanderbilt University, Nashville, Tennessee 37235, United States

## Abstract

Dynamic protein–protein interactions are key drivers of many cellular processes. Determining the relative sequence and precise timing of these interactions is crucial for elucidating the functional dynamics of biological processes. Here, we developed a time-resolved analysis of protein–protein ensembles using a destabilizing domain (TRAPPED) to study protein–protein interactions in a temporal manner. We have taken advantage of a dihydrofolate reductase-destabilizing domain (DHFR­(DD)) that can be fused to a protein of interest and is constitutively degraded by the proteosome. Addition of the ligand trimethoprim (TMP) can stabilize DHFR­(DD), preventing proteasomal degradation of the fusion protein and thereby inducing accumulation in cells. We synthesized and optimized TRimethoprim Analog Probes that maintain stabilization activity and contain a terminal alkyne for Click functionalization and a thiol reactive group to covalently tag DHFR­(DD). Click reaction with a biotin tag and subsequent streptavidin enrichment enable time-resolved mass spectrometric identification of interacting partners. We evaluated the timing of protein interactions of SARS-CoV-2 and SARS-CoV nonstructural protein 15 (nsp15) over a 2 h period. We found interactors GEMIN5 and YBX3, known regulators of SARS-CoV-2 infection that bind viral RNA, as well as CACYBP and FHL1 that implicate nsp15 in the disruption of host ERK1/2 signaling. We further revealed that these interactions remain relatively steady from 0 to 2 h post translation of nsp15. TRAPPED methodology can be applied to determine the sequence and timing of protein–protein interactions of temporally regulated biological processes such as viral infection or signal transduction.

## Graphical abstract

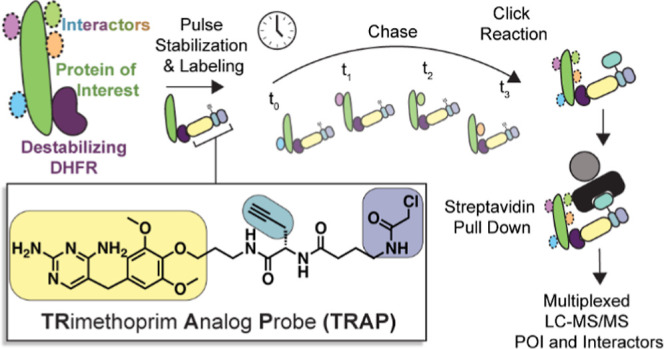

## 1 Introduction

Many biological processes, such as signaling and metabolism, are regulated by protein–protein interactions (PPIs). These interactions are dynamic and constantly changing in response to external stimuli. Many interactions are not binary; rather proteins can have many interacting partners and multiple functions.^1^ Being able to identify when interactions occur with respect to a stimulus or age of a protein can help identify key regulatory steps within complex cellular pathways. Proteomics has become a useful tool to help identify and investigate multifunctional proteins by revealing connections among proteins and pathways.^2,3^ Many viral proteins are multifunctional due to the small genome size of viruses.^4^ Understanding the multiple roles of viral proteins and the timing of these functions via protein–protein interactions can give insight into which interactions are critical and when they take place during infection.

Viral infections are highly regulated by host–virus PPIs. Viral proteins of the pandemic-causing SARS-CoV-2 have been shown to interact with host proteins and protein complexes to perform a variety of proviral functions such as formation of the replication–transcription complex and interferon suppression for host immune evasion.^5–9^ In particular, nonstructural protein 15 (nsp15), the uridine-specific endoribonuclease of SARS-CoV-2, has been shown to modulate the host immune response by cleaving the polyuridine sequence at the 5′ end of negative-strand viral RNA to avoid recognition by RIG-I like receptor MDA5, which activates the type I interferon response.^10–13^ Nsp15 of the related coronavirus strain SARS-CoV from the 2002–2003 epidemic has 88% sequence identity compared to the SARS-CoV-2 homologue and performs the same canonical function during viral infection. A recent study showed that nsp15 also has affinity for transcription regulatory sequences, controlling the synthesis of subgenomic and genomic RNAs that are essential for replication.^14^ Nsp15 may have multiple roles during viral infection, and time-resolved interactomics may shed light on the sequence and timing of key interactions with host factors. While steady-state interactome studies of coronavirus nsp15 have reported key interactions with host factors^6,9,15–18^ and upregulation of genes associated with oxidative phosphorylation and mitochondrial gene expression,^19^ the dynamics of these interactions are poorly understood.

Protein–protein interactions in a native cellular environment have been previously studied using yeast two-hybrid systems, coimmunoprecipitation coupled with Western blot (WB) analysis, and affinity-purification coupled with mass spectrometry (AP-MS).^20,21^ These methodologies have been pivotal in determining steady-state interactions, but they lack the ability to differentiate changes in these interactions over time. Proximity labeling mass spectrometry (BioID & APEX-MS) methods add spatial resolution to the determination of these PPIs.^22–26^ Yet, temporal resolution remains challenging to achieve using these methods due to the inability to synchronize a newly synthesized population of protein. Temporal control can be achieved using unnatural amino acid incorporation or heavy isotope labeling, though these methods are limited to global protein modification instead of one protein of interest.^27,28^ A recent approach using unnatural amino acid incorporation and sequential orthogonal affinity purification for time-resolved interactomics profiling has allowed for the determination of time-resolved interactions for a single protein of interest, though this requires a large amount of material and antibodies for coimmunoprecipitation (co-IP) that recognize the protein of interest.^29^ Small-molecule inhibitors and activators are also powerful tools to specifically control protein regulation. Proteins can be selectively degraded using PROteosomal TArgeting Chimeras, which engage the protein of interest and recruit an E3 ubiquitin ligase, promoting selective degradation of a target protein.^30^ These compounds can provide temporal control for the degradation of a target protein, but no compound that can inversely upregulate the expression of a native target protein has been developed.

Destabilizing domains can be harnessed to provide post-translational regulation of protein upregulation in cells.^31–34^ A protein of interest is fused to an inherently unstable peptide, which leads to constitutive degradation of the fusion protein by the proteasome in the absence of a stabilizing small-molecule ligand. Previously, key mutations in*Escherichia coli* dihydrofolate reductase (ecDHFR) were shown to prevent proper folding of the protein when expressed in human cells.^31^ The resulting destabilizing domain (DHFR­(DD)) can be stabilized by its high-affinity ligand: trimethoprim (TMP). Thus, DHFR­(DD) can be utilized in a gene fusion with a protein of interest for rapid, post-translational control of protein expression.^31^ Destabilizing domains have been successfully applied to control the expression of transcription factors^35,36^ and various other proteins.^33^ Additionally, high selectivity of TMP for ecDHFR over human (hsDHFR) prevents off-target effects, leading to minimal perturbation of the cellular environment.^31,37^

Here, we developed and validated a TRimethoprim Analog Probe (TRAP) that enables time-specific induction of protein expression in mammalian cells by utilizing DHFR­(DD). The optimized TRAP contains three components: a TMP moiety to control DHFR­(DD) accumulation, a terminal alkyne for functionalization via Click chemistry, and a chloroacetamide electrophile that selectively binds a mutant cysteine near the TMP binding site, covalently linking the small molecule to DHFR­(DD) (Figure 1A). We demonstrate that TRAP temporally regulates DHFR­(DD) accumulation and can be enriched using affinity purification. A modified pulse-chase method was implemented in conjunction with TRAP to synchronize and label a newly synthesized population of protein. Our methodology, Time-Resolved Analysis of Protein–protein Ensembles using a Destabilizing Domain (TRAPPED), was used to determine the time-resolved interactome of SARS-CoV-2 and SARS-CoV nsp15, revealing the dynamics of key interactions with host RNA-binding proteins and the ERK1/2 signaling pathway.

**Figure 1:**
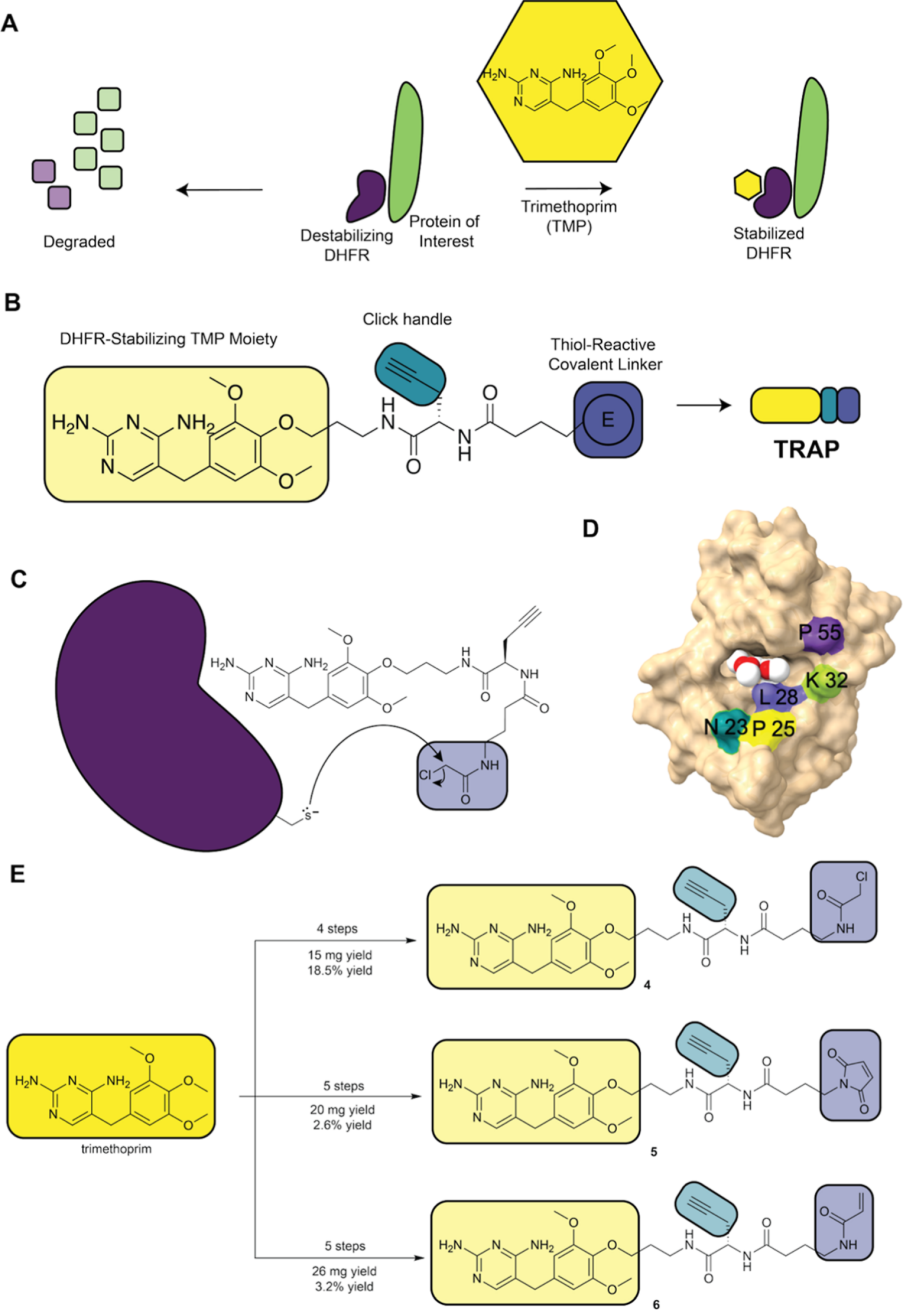
DHFR destabilizing domain can be rescued and labeled with a TRAP: (A) representation of a destabilizing domain. Stabilization of a DHFR­(DD)-POI fusion protein results in rescue from proteasomal degradation when treated with trimethoprim. (B) General structure of trimethoprim-derived probes with three functional domains annotated. The trimethoprim moiety binds and stabilizes DHFR­(DD), a terminal alkyne is included for functionalization via copper-catalyzed alkyne azide Click chemistry (CuAAC), and a thiol reactive linker will covalently react with a mutant cysteine on DHFR. (C) Reaction scheme for the mutant cysteine residue in ecDHFR reacting with the TRAP. (D) A model of ecDHFR (PDB: 6XG5) with trimethoprim bound (red and white space filling) and residues at which engineered cysteines were introduced are indicated. Graphic produced using ChimeraX. (E) Summary scheme for synthesis of TRAPs **4**, **5**, and **6** with chloroacetamide, maleimide, and vinyl ketone groups, respectively.

## 2 Results

### 2.1 Design and Synthesis of a TRimethoprim Analog Probe to Synchronize Protein Accumulation

Trimethoprim (TMP) was functionalized with a terminal alkyne for Click chemistry and an electrophile to covalently bind a cysteine mutation in ecDHFR (Figure 1B). Computational modeling^38,39^ was utilized to predict positions in ecDHFR for which a cysteine residue would be most likely to facilitate covalent binding of a TMP probe by optimizing the distance between the TMP binding site and the engineered cysteine. Since no structure had been solved of TMP bound to ecDHFR at the conception of this project, the model was created by structural alignment of ecDHFR with *Lactobacillus casei* DHFR as previously reported (PDB: 1LUD).^39^ The structure of ecDHFR bound to TMP has since been solved and is shown here (PDB: 6XG5).^40^ We chose five sites (N23, P25, L28, K32, and P55) as the reactive thiol groups were predicted to be within reach of the electrophilic headgroup of the modified TMP probes (Figure 1C,D).^38^ We synthesized three TRAPs with chloroacetamide, maleimide, and vinyl ketone electrophiles (compounds **4**, **5**, and **6** respectively) to form covalent adducts with the cysteine residue (Figure 1E). The distance between the amide bond and the electrophile was optimized to four carbons using analogs of the maleimide TRAP (data not shown). Detailed synthetic schemes and procedures adapted from Jing and Cornish^38^ are included in the Supporting Information (Figure S1A).

### 2.2 TRAP 4 Rapidly and Selectively Binds to DHFR­(DD)^L28C^-YFP

Using a plasmid construct containing an ecDHFR-YFP fusion gene, we introduced the cysteine mutations (N23C, P25C, L28C, K32C, or P55C) via site-directed mutagenesis (SDM) to assess formation of a covalent linkage to the TRAP. Similarly, three destabilizing mutations (R12Y, G67S, and Y100I)^31^ were introduced into ecDHFR to generate the destabilizing domain DHFR­(DD) (Figure 2A). SDM primers are provided in Table S1.

**Figure 2:**
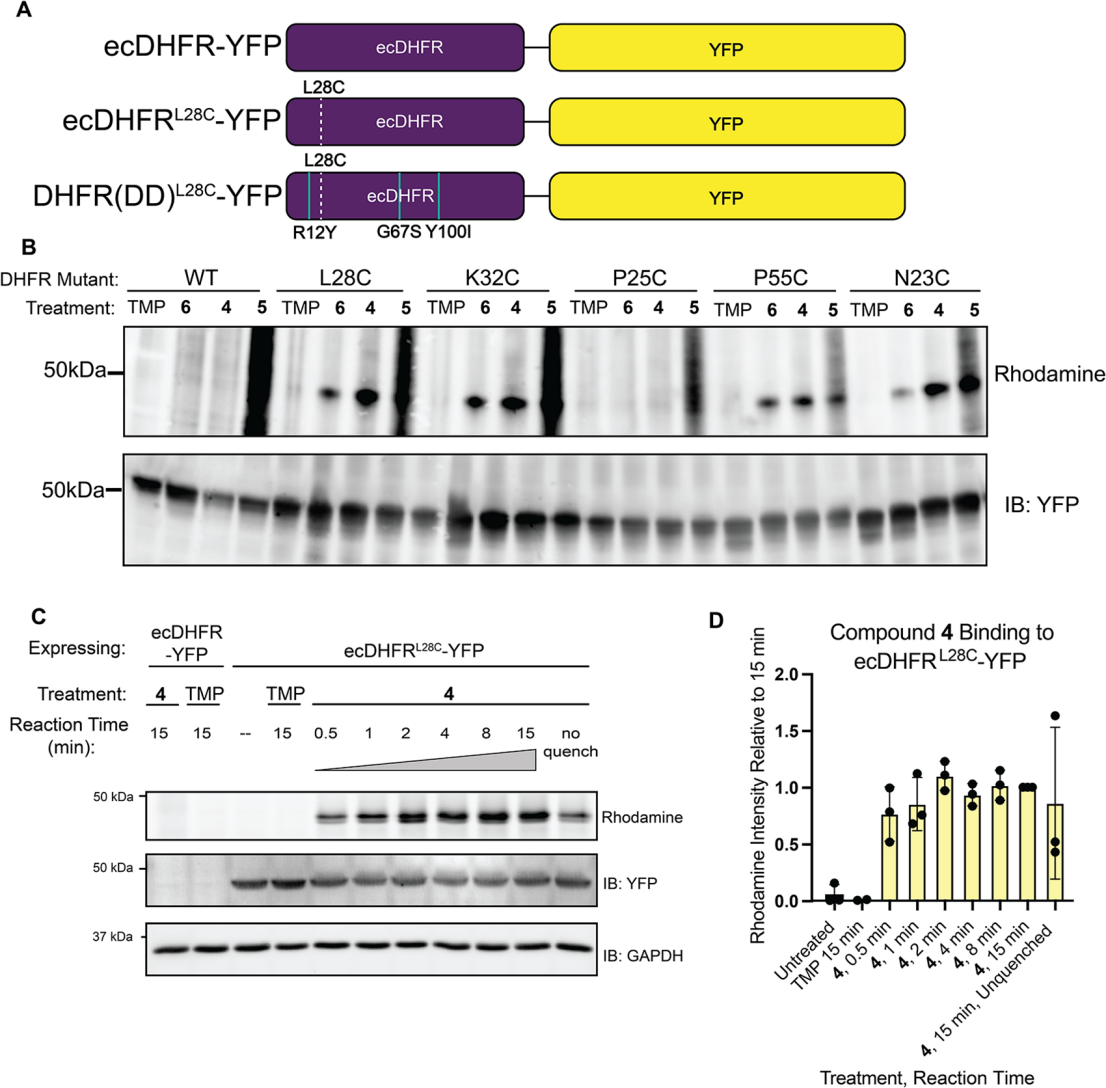
The L28C mutation rapidly and selectively reacts with chloroacetamide TRAP: (A) schematic of the ecDHFR-YFP gene fusions with the L28C mutation for covalent capture (dotted white) and the destabilizing mutations (solid teal) shown in the ecDHFR sequence. (B) WB of lysates from HEK293T cells transfected with ecDHFR-YFP constructs with indicated cysteine mutations and incubated with 10 μM trimethoprim (TMP) or 10 μM TRAP **4**, **5**, or **6** for 4 h. The lysates were then conjugated to TAMRA-Azide-PEG-Desthiobiotin using CuAAC and subjected to SDS-PAGE and WB staining for YFP (using anti-GFP antibody) and rhodamine. Selective reaction of the TRAP with the ecDHFR mutant is indicated by a single rhodamine band as opposed to a streak of a rhodamine signal across many molecular weights. The YFP signal shows that a consistent amount of the ecDHFR mutant was present in each reaction. (C) WB of lysates of HEK293T cells transfected with the ecDHFR^L28C^-YFP construct and reacted with 10 μM TRAP **4** for various lengths of time before being quenched with 1 mM β-mercaptoethanol and conjugated to TAMRA-Azide-PEG-Desthiobiotin using CuAAC. No rhodamine signal is observed in the absence of TRAP **4**, indicating that the TRAP is necessary for labeling. (D) Quantification of the assay from (C). Plot showing the relative intensity of the rhodamine signal of ecDHFR^L28C^-YFP lysates incubated with TRAP **4** for various time points before quenching with β-mercaptoethanol. TRAP **4** is shown to label ecDHFR^L28C^-YFP within 30 s and reach saturation by 2 min.

We next tested the covalent reactivity of the five constructs with TRAPs **4**, **5**, and **6** in parallel using stabilized ecDHFR^XCys^-YFP fusion constructs to determine the effectiveness and selectivity of labeling. The ecDHFR^XCys^-YFP were transiently transfected into HEK293T cells, lysates were harvested and incubated with each probe for 4 h, and a Copper-catalyzed alkyne–azide Click (CuAAC) reaction was performed with tetramethylrhodamine (TAMRA)-Azide-PEG-Desthiobiotin for visualization and enrichment. Upon visualization in WB analysis, the vinyl ketone TRAP (**6**) exhibited low reactivity, the chloroacetamide TRAP (**4**) exhibited moderate reactivity, and the maleimide TRAP (**5**) exhibited high reactivity, as indicated by the strong background rhodamine signal (Figure 2B). Notably, the combination of the L28C mutation and TRAP **4** showed high reactivity and selectivity, as indicated by the high intensity of the rhodamine signal at the ecDHFR-YFP band and the low intensity of the rhodamine signal in WT.

We next measured the rate of covalent labeling of ecDHFR^L28C^ to evaluate the time-resolution. Cell lysates expressing the stabilized ecDHFR^L28C^-YFP were reacted with 10 μM TRAP **4** and quenched with 1 mM β-mercaptoethanol after the specified incubation period. The combination of the ecDHFR^L28C^-YFP and TRAP **4** was shown to label the protein within 30 s and reach saturation by 2 min (Figure 2C,D). In contrast, TRAP **6** required about 4 h to reach saturation of covalent binding to the ecDHFR^P55C^, another pair that showed selective reactivity (Figure S2A). This fast rate of reactivity suggests that TRAP **4** and DHFR­(DD)^L28C^ can be utilized to achieve the synchronization of protein accumulation in cells.

### 2.3 TRAP 4 Rescues and Selectively Tags DHFR­(DD)^L28C^-POI

We next determined the stabilization activity of TRAP **4** on the DHFR­(DD)^L28C^-YFP fusion protein and whether the probe can function in live cells. Briefly, HEK293T cells were transfected with DHFR­(DD)^L28C^-YFP and incubated for specified amounts of time with 10 μM **4**. Lysates were reacted with TAMRA-Azide-PEG-Desthiobiotin. WB showed that the DHFR­(DD)^L28C^-YFP fusion protein was rescued from degradation, as shown by the IB:YFP signal, and also covalently tagged, as shown by the rhodamine signal (Figure 3A). Both the stabilization and covalent capture of DHFR­(DD)^L28C^ by **4** showed a linear increase with time (Figure 3B). We also observed that the relative abundance of the fusion protein was consistently higher in TMP-treated samples compared with samples treated with compound **4**, suggesting that the rescue efficiency of **4** is less than that of the unmodified TMP (Figure S3A). Some background accumulation of the fusion was also observed in the untreated DMSO condition. As this population is not labeled with TAMRA-Azide-PEG-Desthiobiotin, it should not be enriched in downstream streptavidin pull-down and should therefore not contribute to background in future experiments.

**Figure 3:**
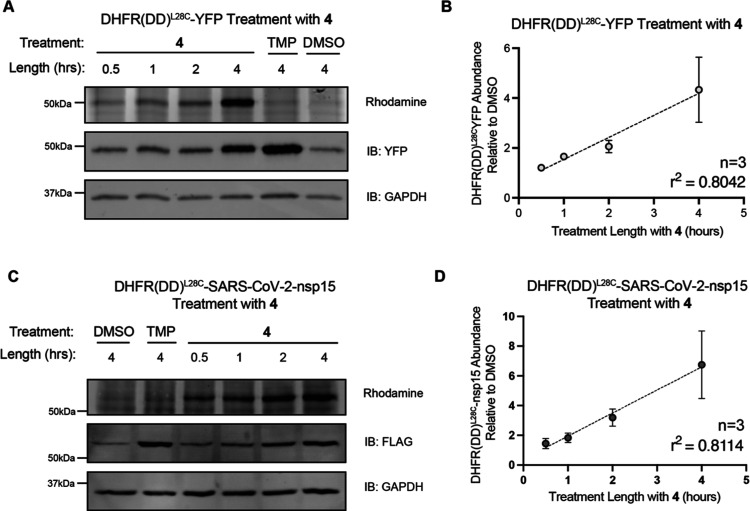
Protein accumulation in vitro can be controlled by TRAP: (A) WB analysis of HEK293T cells transfected with DHFR­(DD)^L28C^-YFP and treated with 10 μM **4**, 10 μM TMP as a positive rescue control, and a negative labeling control or volume equivalent of DMSO as a negative vehicle control. Blots show that accumulation of the protein is time-dependent (IB: YFP), and the labeling capability is limited to lysates rescued by **4** as there is no labeling the above background in the TMP treatment (rhodamine signal). (B) Quantification of YFP abundance relative to DMSO in HEK293T cells transfected with DHFR­(DD)^L28C^-YFP and treated with **4** for increasing time. Simple linear regression was determined for 3 replicates and the coefficient of determination (*R* ^2^) was calculated. (C) WB analysis of HEK293T cells transfected with FT-DHFR­(DD)^L28C^-SARS-CoV-2 nsp15 and treated with 10 μM **4**, 10 μM TMP, or volume equivalent of DMSO. Blots indicate that accumulation of the construct is time-dependent (IB: FLAG), and the labeling capability is limited to lysates rescued by **4** (rhodamine signal). (D) Quantification of FT-DHFR­(DD)^L28C^-SARS-CoV-2 nsp15 abundance relative to DMSO in HEK293T cells transfected with FT-DHFR­(DD)^L28C^-SARS-CoV-2 nsp15 and treated with **4** for increasing time. Simple linear regression was determined for 3 replicates and the coefficient of determination (*R* ^2^) was calculated in GraphPad Prism.

In order to assess whether TRAP **4** can also rescue and label DHFR­(DD)^L28C^ fusions of other proteins of interest, we generated a DHFR­(DD)^L28C^-SARS-CoV-2 nsp15 construct. To reliably analyze the abundance, a FLAG-tag (FT) was appended to the *N*-terminus without any noticeable effects on rescue or labeling compared to the rescue of DHFR­(DD)^L28C‑^YFP (data not shown). The DHFR­(DD)^L28C^-SARS-CoV-2 nsp15 also displayed stabilization and covalent tagging in a time-dependent manner when treated with compound **4** (Figure 3C,D). In addition, the same trend of greater accumulation with TMP treatment compared to compound **4** treatment was observed using the DHFR­(DD)^L28C^-SARS-CoV-2 nsp15 (Figure S3B), though to a lesser extent than that of DHFR­(DD)^L28C^-YFP.

### 2.4 TRAP **4** Can Be Biotinylated and Enriched for Interactomics Characterization

Effective time-resolved interactomics requires that the protein of interest and interacting partners can be enriched. To covalently capture any transient interacting partners, a Dithiobis­(succidimidylpropionate) (DSP) cross-linking reagent was added to cells to nonspecifically capture primary amines within a 12 Å distance. Briefly, DSP was applied to cells immediately prior to lysis to covalently capture interacting partners of the DHFR­(DD) fusion proteins. Next, the lysates were reacted with TAMRA-Azide-PEG-Desthiobiotin and cleaned by methanol/chloroform precipitation. We then performed streptavidin pull-down to isolate TRAP-labeled proteins (Figure 4A).

**Figure 4:**
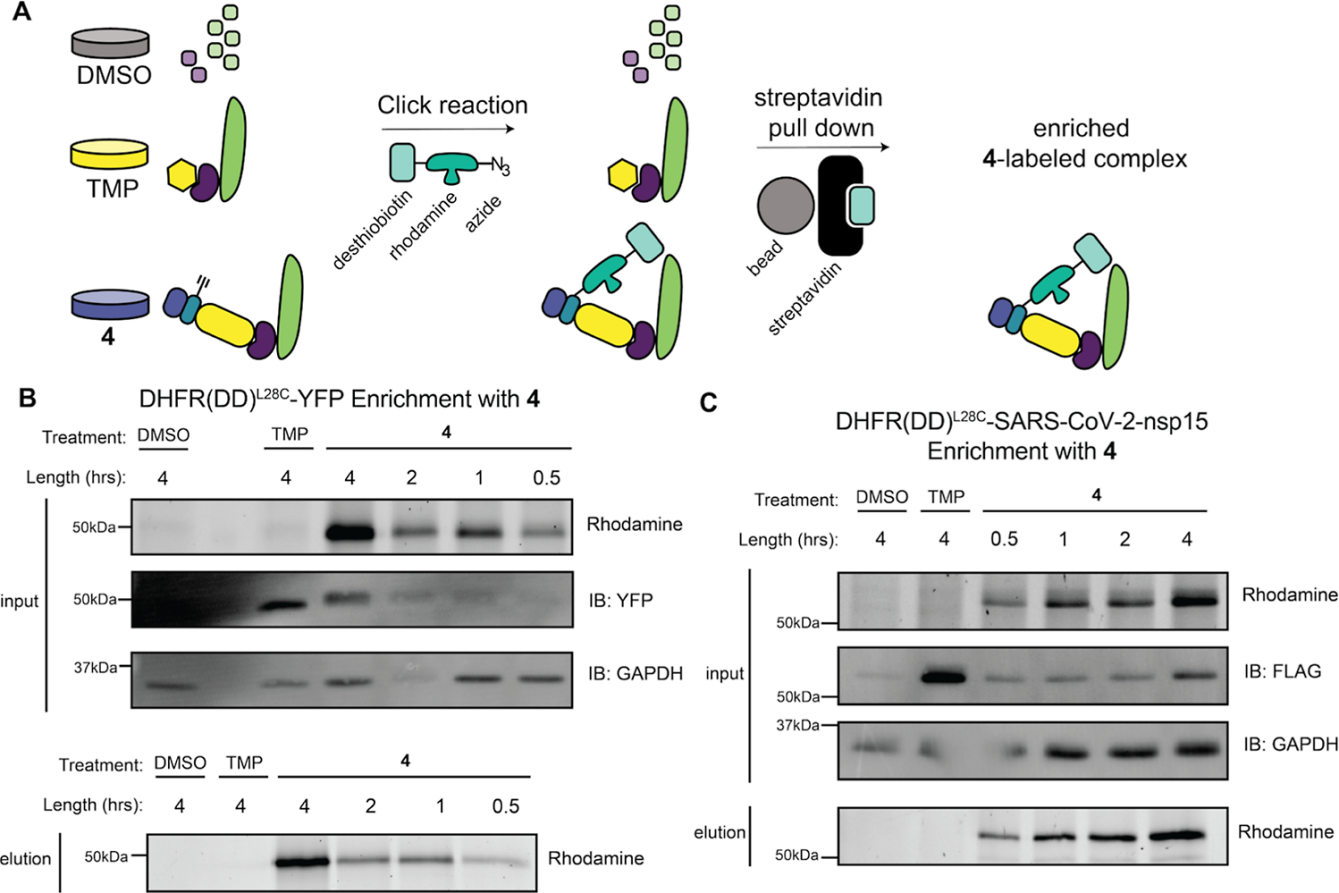
Functionalization of the complex allowed for enrichment of a labeled protein: (A) workflow for DHFR­(DD)^L28C^-POI treatment and affinity enrichment. HEK293T cells were transiently transfected with DHFR­(DD)^L28C^-POI. Cells were then split into separate plates for treatment with DMSO as a negative vehicle control, 10 μM TMP as a positive rescue control, or 10 μM **4** to rescue and label the DHFR­(DD)^L28C^-POI population. A CuAAC reaction was performed to append TAMRA-Azide-PEG-Desthiobiotin containing a rhodamine fluorophore and a desthiobiotin moiety. The DHFR­(DD)^L28C^-POI and interactors were enriched using a biotin–streptavidin pull down resulting in enriched POI. (B) Images of rhodamine and YFP intensity Western blots from DHFR­(DD)^L28C^-YFP transfected HEK293T cells treated with DMSO, 10 μM TMP, or 10 μM **4** before (input) and after affinity purification (elution). (C) Similar images of rhodamine and FLAG intensity Western blots from FT-DHFR­(DD)^L28C^-SARS-CoV-2 nsp15-transfected HEK293T cells treated with DMSO, TMP, or **4** before (input) and after affinity purification (elution).

Using this methodology, DHFR­(DD)^L28C^-YFP (Figure 4B) and FT-DHFR­(DD)^L28C^-SARS-CoV-2 nsp15 (Figure 4C) labeled with **4** were both successfully enriched from lysates while maintaining the time-dependent accumulation observed in unenriched lysates. The presence of the TMP-stabilized constructs in the pull-down input samples and their absence in the elution samples indicate that the fusion protein rescued with TMP is not enriched using streptavidin beads, making this methodology specific for fusion protein bound to compound **4** (Figure S4A,B).

### 2.5 Time-Resolved Interactome of SARS-CoV-2 nsp15 Reveals Steady Interactions with RNA-Binding Proteins

With the stabilization and enrichment strategy for target proteins validated, we turned to determining the time-resolved interactome of nsp15. For this purpose, a modified pulse-chase assay was employed. HEK293T cells transiently transfected with FT-DHFR­(DD)^L28C^-SARS-CoV-2 nsp15 were treated with either DMSO, 10 μM TMP, or 10 μM **4** for 30 min (pulse), which was shown to be sufficient for stabilization and labeling of the DHFR­(DD) complex (Figure S5A). After this 30 min pulse period, 0 h time points for DMSO, TMP, and **4** were cross-linked with DSP to covalently bind nearby proteins and harvested (Figure 5A). The remaining cells pulsed for 30 min with **4** were exposed to media containing 10-fold excess TMP during the chase period to compete off any unbound probe molecule. Cells were then cross-linked with DSP and harvested in increments of 30 min up to 2 h into the chase period. One set of cells was treated continuously with **4** for the course of the experiment (2.5 h total) to capture the steady-state interactions of the complex and to act as a booster channel in the mass spectrometry tandem mass tag (TMT) multiplexing. WB analysis showed that the population of protein rescued and labeled during the 30 min pulse period maintains steady abundance during the chase period, indicating that the labeled population of protein can be monitored over time without substantial degradation of the labeled protein or labeling outside of the 30 min pulse (Figure 5B,C).

**Figure 5:**
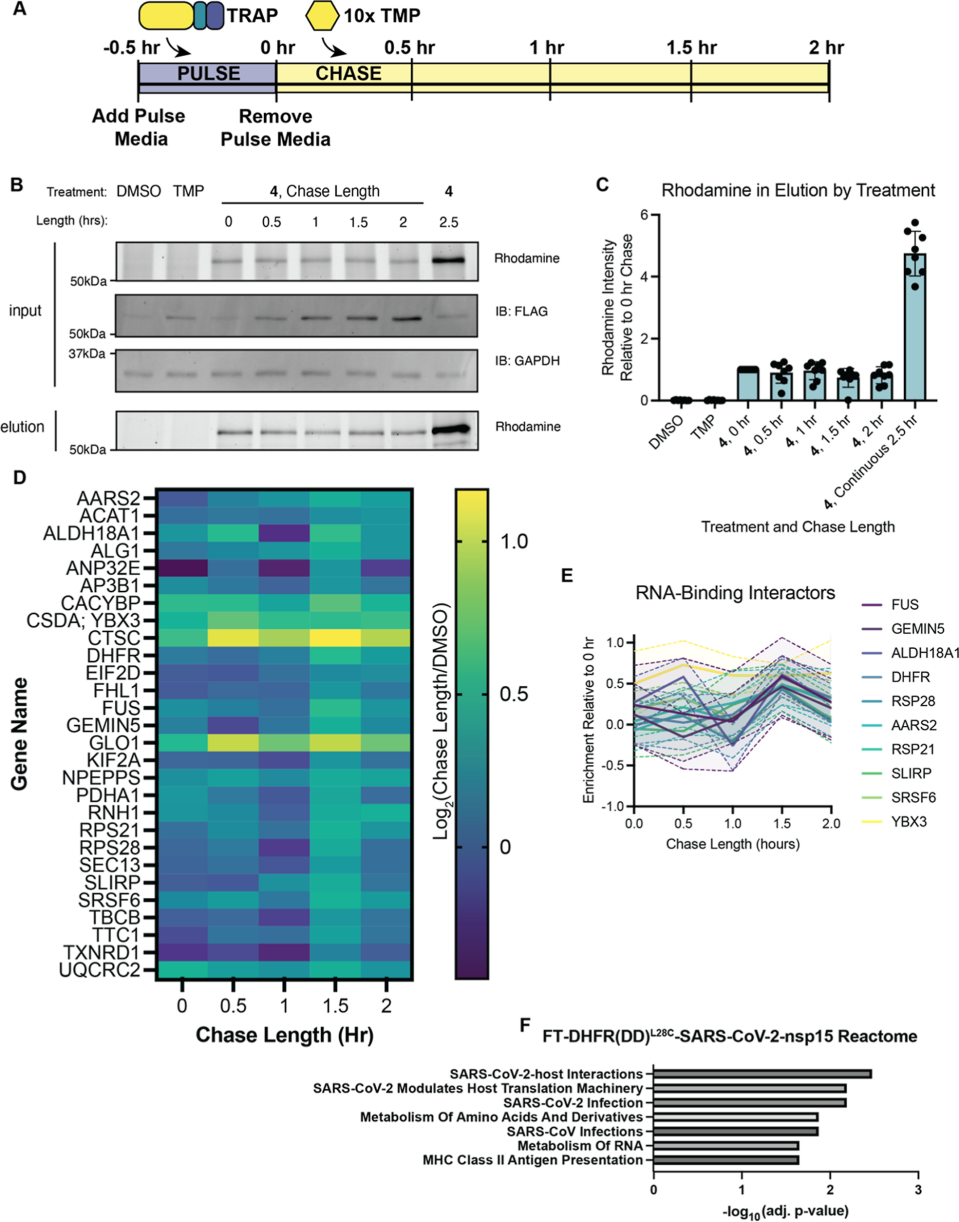
SARS-CoV-2 nsp15 has interactions with RNA-binding proteins that remain steady 2 h after translation: (A) timeline of the pulse-chase assay used to collect time-resolved interactomics data for nsp15. (B) Images of rhodamine, FLAG, and GAPDH signals in Western blots from FT-DHFR­(DD)^L28C^-SARS-CoV-2 nsp15-transfected HEK293T cells treated with 10 μM **4** at the indicated chase time points before (input) and after affinity purification (elution). DMSO and TMP controls are included as negative controls for the enrichment. (C) Quantification of rhodamine intensity in elution SDS-Page gels (from C) relative to the 0 h chase sample for 8 replicates of TRAPPED. Error bars indicate standard error of the mean (SEM). (D) Heat map showing the change in interaction strength of each FT-DHFR­(DD)^L28C^-SARS-CoV-2 nsp15 interactor. Log_2_(fold change) compared to the DMSO negative control channel is shown with respect to the color bar key for each time point from 0 to 2 h after a 30 min pulse of TRAP **4**. (E) Enrichment traces relative to DMSO background of RNA-binding proteins that interact with SARS-CoV-2 nsp15. The solid line connects the mean at each time point during the chase period and the dashed line with shaded region represents the SEM (F) Gene Ontology (GO) term analysis searching against the Reactome database reveals overlap between nsp15 interactors identified in this study and known SARS-CoV-2 host interactions.

Following affinity enrichment of the cross-linked lysates reacted with TAMRA-Azide-PEG-Desthiobiotin, samples were reduced, acetylated, trypsin-digested, labeled with TMTpro 16-plex reagents, and analyzed using LC–MS/MS (Tables S2 and S3). After median normalization (Figure S5B,C), TMT abundances from MS2 spectra were used to compile a list of interactors by comparing the continuous sample to DMSO and TMP negative controls (Figure S5D,E). The changes in abundance over the pulse-chase assay were then determined for the interactors and plotted in a heat map to visualize the changes in interactors over time (Figure 5D and Table S6). Generally, the interaction strength between SARS-CoV-2 nsp15 and its interactors stayed relatively constant throughout the chase period, though there are some exceptions like GEMIN5, which is less enriched at 0.5 h and more enriched at 1.5 h. The lack of interactor abundance changes may be due to the expression system in the absence of viral RNA that is necessary for nsp15 endonuclease activity.

When the MS data were searched for previously identified interactors^6,9,15–18^ from BioID and AP-MS studies in HEK293T cells and A549 cells, 2 proteins were identified as interactors of SARS-CoV-2 nsp15 in our data set: CACYBP and FHL1 (Figure S5F). These two proteins have been implicated as anti- and pro-viral factors, respectively, but have not been explored in the context of coronaviruses.^41,42^

GO term enrichment analysis was performed using Enrichr to determine trends in biological function.^43^ Many of the identified interactors are involved in RNA binding including: DHFR, RPS28, AARS2, FUS, GEMIN5, ALDH18A1, SLIRP, SRSF6, YBX3, and RPS21. Searching the interactors against the Reactome 2022 database also revealed a strong association with known SARS-CoV-2 host interactors, including SEC13, RPS28, GEMIN5, and RPS21 (Figure 5F and Table S5). Nearly all of the 29 interactors of SARS-CoV-2 nsp15 were also found to be associated with cytosolic subcellular localization when analyzed with SubcellulaRVis, indicating that the fusion protein is localizing as expected for SARS-CoV-2 nsp15 (Table S4).^44^

### 2.6 Time-Resolved Interactome of SARS-CoV nsp15 Reveals Translation Interactors

Next, time-resolved interactions were determined for SARS-CoV nsp15 using the TRAPPED methodology (Figure 6A, Tables S7, S8, and S10). Despite SARS-CoV and SARS-CoV-2 sharing 88% sequence identity and having the same canonical function, SARS-CoV nsp15 counteracts type I interferon (IFN) induction 32 times more than SARS-CoV-2.^45^ Identifying strain-specific interactions can help explain this difference in host immune suppression. While the amount of the SARS-CoV-2 nsp15 fusion protein remained constant during the chase period, the SARS-CoV nsp15 fusion protein showed a slight decrease in abundance during the chase period, possibly due to incomplete stabilization of the fusion or other avenues of degradation (Figure S6A,B). Interactors of SARS-CoV nsp15 were identified by comparing the continuously treated sample to the negative vehicle control (Figure S6C), are also localized to the cytoplasm (Table S9), and are associated with translation initiation and components of the large ribosomal subunit (Figure 6B,C). Interactions with translation initiation factors remain constant throughout the time course, while interactions with members of the ribosome decrease at 1.5 h before rising again at 2 h. CACYBP was identified as an interactor in both the SARS-CoV and SARS-CoV-2 data sets and maintains stable enrichment over the course of 2 h, though there is some variation in the later time points (Figure 6D).

**Figure 6:**
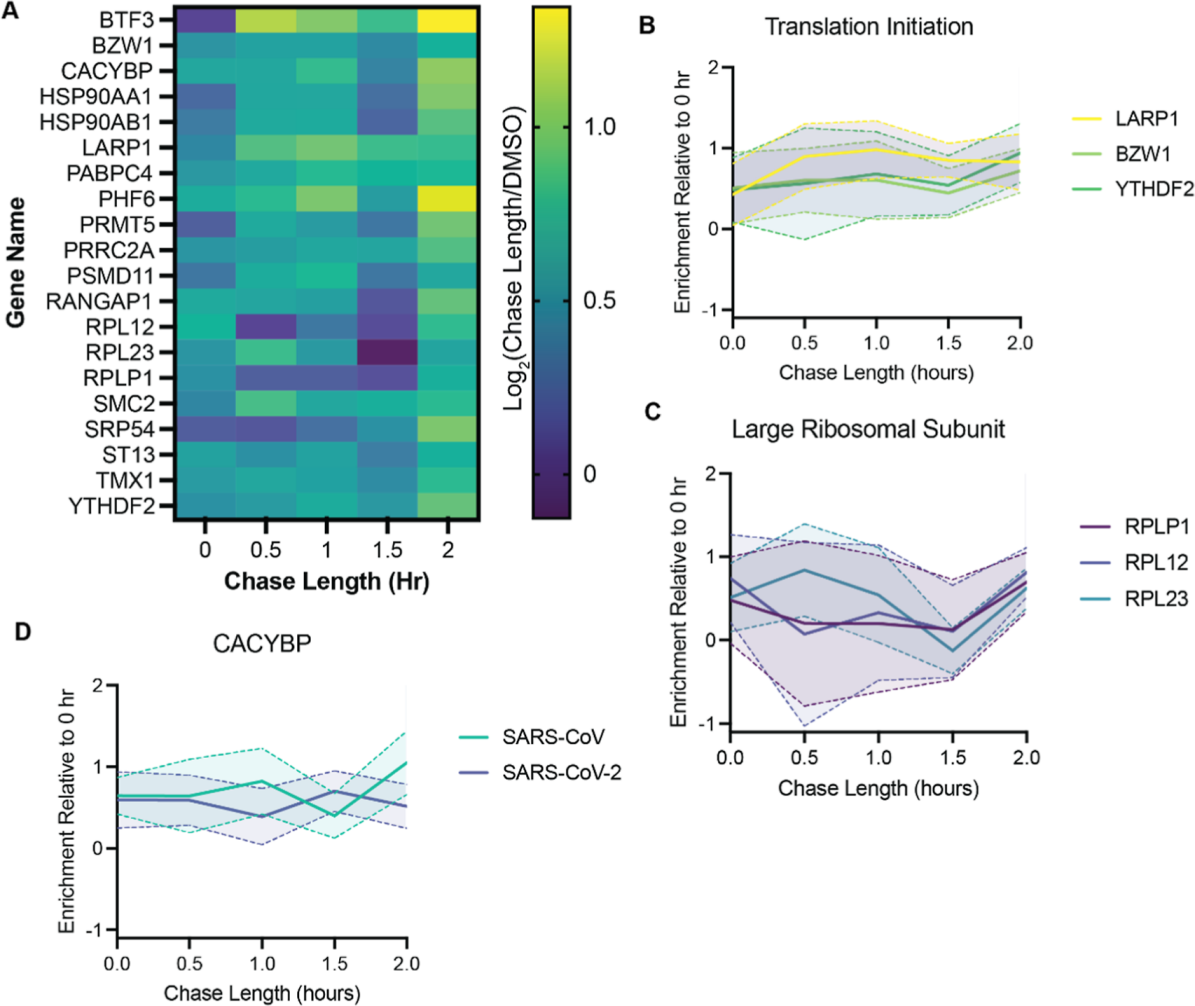
SARS-CoV nsp15 interacts with the translation machinery. (A) Heat map showing the change in interaction strength of each FT-DHFR­(DD)^L28C^-SARS-CoV-nsp15 interactor throughout the chase time course for 6 replicates of TRAPPED. Log_2_(fold change) compared to the DMSO negative control channel is shown. (B) Enrichment traces relative to DMSO background of translation initiation proteins that interact with SARS-CoV nsp15. The solid line connects the mean at each time point during the chase period and the dashed line with the shaded region represents the SEM (C) Enrichment traces relative to DMSO background of large ribosomal subunits that interact with SARS-CoV nsp15. (D) Comparison of CACYBP enrichment with SARS-CoV and SARS-CoV-2 nsp15 throughout the chase time course.

## 3 Discussion

Building upon previous studies of destabilizing domains, we developed a trimethoprim analogue probe (TRAP) that enables temporal control of protein accumulation and selective enrichment for interactomics studies. We performed a screen of cysteine locations and TRAP electrophiles and found that chloroacetamide and vinyl ketone TRAPs were selective for mutant DHFR while the maleimide TRAP exhibited more promiscuous labeling. While many cysteine–electrophile pairs were selective, L28C and the chloroacetamide TRAP **4** were shown to bind rapidly, and therefore this optimized pair was selected for use in TRAPPED methodology. While TRAP **4** binding to DHFR^L28C^ was shown to be rapid in a cell-free lysate system, detectable accumulation in cells required 30 min. This discrepancy may be due to the cellular permeability of TRAP or the translation rate of new proteins. Understanding the kinetics that contribute to slower binding in cells will require further investigation. Even though the native ligand TMP binds to DHFR with higher affinity than TRAP molecules, the covalent binding of TRAP to DHFR is necessary to facilitate efficient enrichment of the DHFR-fusion and cross-linked interaction partners. TRAP **4** can control the accumulation of a DHFR­(DD)^L28C^-POI fusion in a time-dependent manner. Functionalization of **4** using TAMRA-Azide-PEG-Desthiobiotin via CuAAC allowed the protein complex to be visualized by a rhodamine fluorophore and enriched via a streptavidin pull-down. These same characteristics were maintained when an FT-DHFR­(DD)^L28C^-SARS-CoV-2 nsp15 fusion was used.

We implemented a pulse-chase assay to selectively label a population of FT-DHFR­(DD)^L28C^-SARS-CoV-2 nsp15 and monitor the interactions for the following 2 h to determine time-dependent changes in the interactome of SARS-CoV-2 nsp15. These time-resolved interactomics data revealed interactions that remain relatively constant throughout the chase period. These data suggest that nsp15 interactions are not highly dynamic. One limitation of our study is that we conducted the interaction time courses in the absence of viral RNA, which may limit time-dependent changes. We expect that TRAPPED conducted in the presence of viral RNA or infectious virus would reveal dynamic interactions reflecting the multiple functions of nsp15. In addition, TMP washout during the chase period, which was intended to prevent labeling of nsp15 with **4** after the 30 min pulse, continued to stabilize newly synthesized nsp15 during the chase period. Although this TMP-stabilized, unlabeled population of nsp15 was not enriched, the continued accumulation of the protein of interest may have affected proteostasis or other biological processes associated with degradation or nsp15 function. Zero-hour samples that did not undergo a TMP washout may have lengthened stabilization compared to the remaining time points, which could bias interactions.

Most of the interactors of SARS-CoV-2 nsp15 that were identified in this study have not been reported previously, although interactions between two proteins identified here have been shown to interact with SARS-CoV-2 nsp15: FHL1 and CACYBP. The small amount of overlap between this study and previous studies may be attributed to the steady-state nature of previous studies or to different methods of data acquisition, for example, the absence of DSP cross-linkers in other studies. Though previously identified interactors of nsp15 were not enriched in our data set, the detection of some of these proteins indicates that they are at least present in the sample and may need to have more nsp15 present in order to interact at a detectable level.

The calcyclin-binding protein (CACYBP) has several cellular functions including dephosphorylation, ubiquitination, and involvement in cytoskeletal dynamics.^46^ In particular, CACYBP binds and dephosphorylates ERK1/2, which is involved in many signaling pathways including cell proliferation and stress.^47^ Other researchers have shown that the EGFR/ERK signaling is activated by the Spike protein of SARS-CoV-2 and inhibition of this signaling has a pro-viral effect.^48^ The interaction between nsp15 and CACYBP identified here could possibly interfere with host ERK1/2 signaling dynamics to increase the efficiency of viral proliferation. Four-and-a-half LIM domain protein 1 (FHL1) is mostly involved in muscle function and development but has been shown to play a key role in chikungunya virus infection via interaction with nsP3.^42^ FHL1 also has a well-known role in activating ERK1/2 signaling through interaction with transcription factor SP1 ^49^ and through direct interaction with ERK1/2 signaling components.^50^ Further investigation into the roles of SARS-CoV-2 and SARS-CoV nsp15 in ERK1/2 signaling may reveal key regulatory mechanisms during viral infection.

RNA-binding proteins were identified as a prominent category of interaction partners of SARS-CoV-2 nsp15. Germ-associated protein 5 (GEMIN5) has been described as an antiviral factor in Sindbis virus by binding the 5′ cap and UTR of viral RNA to suppress viral translation.^51^ GEMIN5 also has an antiviral effect on SARS-CoV-2, possibly by a similar cap-binding mechanism.^52^ Our data suggest that it may coordinate with nsp15, which binds to the 5′-UTR region of the complementary negative-sense RNA strand. Y-Box binding protein 3 (YBX3) is another RNA binding protein that is known to bind the 3′ UTR of mRNA transcripts to increase stability.^53^ It has recently been shown that YBX3 is necessary for SARS-CoV-2 viral replication.^54^ The interaction between nsp15 and YBX3 remains relatively constant during the time course, indicating that this interaction forms quickly after nsp15 is translated and is maintained as nsp15 ages. Any regulatory function of the interaction between nsp15 and YBX3 has not yet been explored, though these two proteins both bind the 3′ region of RNA transcripts, and nsp15 may also play a regulatory role in the proviral effects of YBX3.

Some limitations must be noted for the TRAPPED methodology. TRAPPED builds upon steady-state interactomics methodology to add temporal information to the interaction network, although the interactions do not necessarily require TRAPPED to be detected. Background accumulation of the destabilizing domain can occur in the absence of a ligand due to presence of too much transfected DNA that can overwhelm the proteasome^55^ or due to low levels of ligand in the media serum. We found that the background was reduced in this study with the use of a serum lot that contained minimal amounts of antibiotics (data not shown). In our hands, destabilizing DHFR was not able to be stabilized by TMP in the case of all DHFR­(DD)-POI fusion proteins. Some optimization may be required to develop a destabilizing domain-POI pair. This limitation may be addressable using optimized destabilizing mutations in DHFR that were discovered after the conception of this project.^56^

This work presents an adaptation on previously developed methodology to reveal the temporal landscape of protein–protein interactions.^31,38^ We optimized and synthesized probe analogs of trimethoprim that selectively and rapidly bind a destabilizing domain that allowed for synchronization and labeling of a population of protein. The complex can be functionalized with Click chemistry and enriched with interacting proteins. A time-resolved method, TRAPPED, was used to determine the relative timing of key interactions with two homologues of coronavirus nsp15, revealing key interactions with RNA-binding proteins and the ERK1/2 signaling pathway. The characterization of TRAPPED here demonstrates that the method can be applied to proteins with multiple functions to determine when each function occurs through the characterization of interaction partners. TRAPPED can also be utilized to investigate interactions with protein folding machinery and protein transport machinery shortly following translation. Similar temporal protein interactomics studies have been used to study how interactions with proteostasis factors and secretory factors differ between WT and disease states.^29^ Overall, TRAPPED is a widely applicable chemical biology tool to study the timing of protein–protein interactions in cells.

## 4 Methods

### 4.1 Synthesis and Characterization of Probe Compounds

Detailed information on the reagents and procedures used in the synthesis of the probe compounds can be found in the Supporting Information. The identity of the final probe compounds was confirmed by ^1^H and ^13^C NMR and high-resolution mass spectrometry (Indiana University Mass Spectrometry Core). These spectra are included in the Supporting Information.

### 4.2 Plasmid Generation

The parent plasmid containing DHFR­(DD)-YFP was generously provided by Shoulders et al.^35^ We utilized this construct to create a plasmid containing stabilized ecDHFR-YFP using the NEBuilder HiFi DNA Assembly kit and the Q5 polymerase. Five individual rounds of mutagenesis yielded the five ecDHFR-YFP variants with additional Cys mutations: N23C, P25C, L28C, K32C, P55C. The forward and reverse primers for SDM to introduce these cysteine mutations were the same for the ecDHFR and DHFR­(DD) mutants and are listed in Table S1. PCRs were performed using a Pfu Turbo polymerase (Agilent) following the manufacturer’s instructions for L28C, K32C, and P55C variants. The temperature for denaturation was 60 °C and the extension time used was 8 min. Products amplified with Pfu Turbo were digested with 1 μL of DpnI for 2 h and transformed into DH5α *E. coli* cells (NEB). Q5 polymerase was used for N23C and P25C variants with an annealing temperature of 65 °C and extension time of 7 min. Products amplified with Q5 were digested with 1 μL of DpnI for 2 h, phosphorylated with T4 polynucleotide kinase, and ligated with T4 DNA ligase before being transformed into DH5α *E. coli* cells (NEB). The plasmids were purified by a Qiagen Plasmid Plus Midiprep kit, and correct DNA sequences were confirmed by Sanger sequencing performed by GENEWIZ.

SARS-CoV-2 nsp15 was amplified from pLVX-EF1alpha-SARS-CoV-2 nsp15-2xStrep-IRES-Puro (AddGene 141381)^6^ using Q5 polymerase according to the manufacturer’s protocol with an annealing temperature of 63.9 °C and an extension time of 30 s with primers listed in Table S1 (SARS_CoV2_nsp15_F and R). The vector containing FT-DHFR­(DD)^L28C^ was also amplified using Q5 polymerase with an annealing temperature of 63.5 °C and an extension time of 3.5 min with primers listed in Table S1 (DHFR­(DD)_L28C_nsp15_F and R). Products were digested with DpnI and HiFi assembly was performed according to manufacturer’s protocol with a 1:5 ratio of the vector/insert. The sequence was confirmed via Sanger sequencing using a CMV_F universal primer.

### 4.3 Cell Culture and Transfections

All cell culture reagents were purchased from Corning unless otherwise noted. HEK 293T cells were cultured in Dulbecco’s Modified Eagle Medium (DMEM) with 10% v/v fetal bovine serum, 1% v/v 100× penicillin/streptomycin, and 1% v/v 100× glutamine. All cells were maintained at 37 °C under 5% CO_2_. Cells were plated in 10 cm dishes at 2 × 10^6^ cells/dish for 24 h before transfecting with 5 μg DNA per plate using a calcium phosphate transfection method. 18 h after transfecting, the media was removed, and the plates were washed with 5 mL of phosphate buffered saline (PBS) before being replaced with 10 mL of fresh DMEM. When no direct cell treatments were performed, the cells were harvested 18–24 h after exchanging the media.

For direct cell labeling experiments, plates were passaged at a 1:6 dilution into 6-well plates 18–24 h after exchanging the DMEM. The cells were treated with DMSO, TMP, or one of the TRAP compounds as a 1000X stock solution (stock solution concentration 10 mM in DMSO). After the designated amount of time, the cells were immediately harvested.

### 4.4 Pulse-Chase Assay

HEK293T cells were seeded at 3.75 × 10^6^ total cells in a 15 cm dish and transfected as described above with 12.5 μg of the respective plasmid. After exchanging the media, cells were incubated for 1 h and then passaged 1:9 into 8 × 6 cm dishes coated with poly-d-lysine hydrobromide (Sigma-Aldrich, P64075 mg) according to manufacturer’s instructions. After 4 h, the media was aspirated and replaced with media containing 10 μM TMP, 10 μM **4**, or the respective volume of DMSO as a vehicle control. After 30 min of incubation, the zero-hour time points were collected and all other cells were washed with 1 mL of media containing 100 μM TMP or 1 mL of DMSO media (continuous sample only). The samples were then incubated for the corresponding amount of time in 100 μM TMP media or 10 μM **4** media (continuous sample only) and harvested using DSP cross-linking and lysis (below).

### 4.5 DSP Cross-Linking

Cell samples to proceed through streptavidin pull-down were subjected to cross-linking using dithiobis­(succinimidyl propionate) (DSP) (Fisher, PI22585) to maintain transient interactions due to stringent wash conditions during the affinity enrichment. Before splitting cells into experimental plates, the plates were coated with poly-d-lysine hydrobromide (Sigma-Aldrich, P64075 mg) to adhere cells to the plate. After treatment and prior to harvesting, adhered cells were washed twice with PBS at RT. DSP was dissolved in DMSO for a 50 mM 100x stock immediately before use. DSP was diluted 100x in 1 mL of PBS for a working concentration of 0.5 mM, added directly to cells, and incubated for 10 min at 37 °C. The DSP was then quenched with 100 μL of 1 M Tris pH 7.5 and incubated for 5 min at 37 °C.

### 4.6 Cell Harvesting and Lysis

For cell samples that were not directly treated, the cells were harvested by placing the 10 cm dishes on ice, aspirating the media, and washing each plate with 5 mL of cold PBS. Cells were scraped in 1 mL of PBS + 1 mM EDTA and transferred into 1.5 mL microcentrifuge tubes on ice. The cells were pelleted at 400*g* for 15 min, washed once in 1 mL of cold PBS, and pelleted again. The cells were lysed in 1 mL of cold Radioimmunoprecipitation assay buffer (RIPA, 150 mM NaCl, 1% v/v Triton X-100, 0.5% w/v sodium deoxycholate, 0.1% w/v SDS, 50 mM Tris pH 7.5) with a cOmplete EDTA-free protease inhibitor (Sigma-Aldrich, 4693159001). The lysate suspensions were incubated on ice for 30 min before being centrifuged at 21,100*g* for 5 min at 4 °C. The supernatant was collected, and the protein concentration in the cleared lysates were normalized to 1.0 mg mL^–1^ using the Pierce BCA Protein Assay (Thermo, 23225) and dilution with RIPA buffer.

For cell samples that were directly treated with compounds, the cells were harvested by placing the 6-well dishes on ice, aspirating the media, and washing each well with 1 mL of cold PBS. A 200 μL portion of RIPA with the cOmplete EDTA-free protease inhibitor was added to each well to lyse the cells directly on the plate. The plates were incubated with RIPA buffer for 15 min on ice before transferring the contents of each well into a fresh 1.5 mL microcentrifuge tube. The lysates were cleared at 21,100*g* for 5 min at 4 °C, and then the protein concentrations were normalized to 1.0 mg mL^–1^ using the Pierce BCA Protein Assay (Thermo, 23225) and dilution using RIPA buffer.

### 4.7 Click Reaction, Gel Electrophoresis, and Western Blotting

A Click reaction “master mix” solution was freshly prepared with 1.2 μL per sample 20 mM Cu_2_SO_4_ (0.8 mM final), 1.2 μL per sample 40 mM BTTAA (1.6 mM final, Click Chemistry Tools, 1236), 1.5 μL per sample 100 mM sodium ascorbate (5 mM final), and 0.6 μL per sample 5 mM TAMRA-Azide-PEG-Desthiobiotin (100 μM final, BroadPharm, BP-22475). For each sample, 25.5 μL of the lysates normalized to 1.0 mg mL^–1^ was transferred to a fresh microcentrifuge tube and mixed with 4.5 μL of the reagent master mix. The samples were incubated at 37 °C shaking at 750 rpm for 1 h. The sample was mixed with 6X SDS loading buffer and heated to 95 °C in a heat block for 5 min. The samples were then loaded into a freshly prepared 10% SDS-PAGE gel and run for 1 h at 200 V. The gels were transferred to PVDF membranes (Millipore Sigma, IPFL00010) utilizing either the TransBlot Turbo (BioRad) by the manufacturer’s instructions or by wet transfer at 100 V for 80 min.

The blots were then immediately imaged for a fluorescence signal (rhodamine or Cy3). All images were obtained on a BioRad ChemiDoc MP imaging system. The blots were then blocked with 5% w/v milk in Tris-buffered saline and 0.01% Tween-20 (TBST) for 1 h at RT. After three rinses with TBST, the blots were probed with the appropriate primary antibody at 4 °C for 16 h or at RT for 1 h (anti-FLAG: Sigma-Aldrich #F1804) (anti-GFP: Vanderbilt Antibody and Protein Resource Core clone #1C9A5) (anti-GAPDH, GeneTex #GTX627408) and StarBright fluorescent antimouse (BioRad, 12004158) antibody at 4 °C for 1 h. Quantification of the signal from gels and Western blots was conducted using Image Lab (Bio-Rad). Lanes were defined using the lane tool, bands were selected using the band tool, and lane profiles were adjusted to contain the entire signal above the background. The total adjusted band volume was then normalized to a background control and a positive control.

### 4.8 Streptavidin Pull-down

Lysates normalized to 1.0 mg mL^–1^ were prepared for streptavidin pull-down by performing the Click reaction described previously on a 200 μL total volume scale. After the 37 °C incubation step, the samples were mixed with 300 μL of methanol, 100 μL of chloroform, and 300 μL of water and vortexed briefly. The samples were spun down, the methanol/water layer was removed, and another 500 μL of methanol was added. This caused the protein to precipitate as a pellet, and the samples were spun; the supernatant was removed from the pellets. The pellets were dried and redissolved in 100 μL of 6 M urea and 1% w/v SDS in PBS, and the samples were diluted to a total volume of 1.1 mL with PBS.

50 μL of slurry per sample of prewashed streptavidin resin (Thermo Fisher, 20359, Pierce High-Capacity Streptavidin Resin) was added to each sample, and the samples were incubated rotating at RT for 1 h. The beads were pelleted by centrifugation, and the supernatant was removed. The beads were washed with 400 μL of 1% w/v SDS in PBS six times before adding 100 μL of elution buffer (50 mM biotin, 1% w/v SDS in PBS) and incubating at 95 °C for 5 min before pelleting the resin and retrieving the supernatant containing the eluted proteins. This elution step was repeated with another 100 μL of elution buffer, and the supernatants were combined. The elution samples were then subjected to SDS-PAGE as previously described, and the gels were imaged for rhodamine fluorescence. Elution samples were subjected to methanol chloroform precipitation as described previously and were subsequently prepared for mass spectrometry analysis.

### 4.9 Mass Spectrometry Sample Preparation

Protein pellets were resuspended in 3 μL of 1% w/v Rapigest SF Surfactant (Waters, 186002122) followed by the addition of 10 μL of 50 mM HEPES pH 8.0 and 34.5 μL of water. Samples were reduced with 5 mM tris­(2-carboxyethyl)­phosphine (TCEP) (Sigma, 75259) at RT for 30 min and alkylated with 10 mM iodoacetimide (Sigma, I6125) in the dark at RT for 30 min. Proteins were digested with 0.5 μg of trypsin/Lys-C protease mix (Pierce, A40007) by incubating for 16–18 h at 37 °C and shaking at 750 rpm. Peptides were reacted with TMTpro 16plex reagents (Thermo Fisher, 44520) in 40% v/v acetonitrile and incubated for 1 h at RT. Reactions were quenched by the addition of ammonium bicarbonate (0.4% w/v final concentration) and incubated for 1 h at RT. TMT-labeled samples were then pooled and acidified with 5% v/v formic acid (Fisher, A117). Samples were concentrated using a speedvac and resuspended in buffer A (97% water, 2.9% acetonitrile, and 0.1% formic acid, v/v/v). The cleaved Rapigest SF surfactant was removed by centrifugation for 30 min at 21,100*g*.

### 4.10 Liquid Chromatography–Mass Spectrometry

Multidimensional Protein Identification Technology (MudPIT) microcolumns were prepared as previously described.^57^ Peptide samples were directly loaded onto the columns using a high-pressure chamber. Samples were then desalted for 30 min with buffer A (97% water, 2.9% acetonitrile, 0.1% formic acid v/v/v). LC–MS/MS analysis was performed using an Exploris480 (Thermo Fisher) mass spectrometer equipped with an Ultimate3000 RSLCnano system (Thermo Fisher). MudPIT experiments were performed with 10 μL sequential injections of 0, 10, 30, 60, and 100% buffer C (500 mM ammonium acetate in buffer A), followed by a final injection of 90% buffer C with 10% buffer B (99.9% acetonitrile, 0.1% formic acid v/v), and each step was followed by a 92 min gradient from 5% to 35% B and a short column flush up to 85% B for 7 min with a flow rate of 500 nL/min on a 20 cm fused silica microcapillary column (ID 100 μm) ending with a laser-pulled tip filled with Aqua C18, 3 μm, 125 Å resin (Phenomenex). Electrospray ionization (ESI) was performed directly from the analytical column by applying a voltage of 2.2 kV with an inlet capillary temperature of 275 °C. Data-dependent acquisition of mass spectra was carried out by performing a full scan from 400 to 1600 *m*/*z* at a resolution of 120,000. Top-speed data acquisition was used for acquiring MS/MS spectra using a cycle time of 3 s, with a normalized collision energy of 36, 0.4 *m*/*z* isolation window, automatic maximum injection time, and 100% normalized AGC target, at a resolution of 45,000 and a defined first mass (*m*/*z*) starting at 110.

Peptide identification and TMT-based protein quantification were carried out using Proteome Discoverer 2.4. MS/MS spectra were extracted using the Thermo Xcalibur.raw file format and searched using SEQUEST against a Uniprot human proteome database (accessed 03/2014 and containing 28,860 entries) supplemented with the appropriate coronavirus nsp15 gene. The database was curated to remove redundant protein and splice-isoforms. Searches were carried out using the following parameters: 20 ppm peptide precursor tolerance, 0.02 Da fragment mass tolerance, minimum peptide length of 6 amino acids, trypsin cleavage with a maximum of two missed cleavages, dynamic methionine modification of +15.995 Da (oxidation), dynamic protein N-terminus +42.011 Da (acetylation), −131.040 (methionine loss), −89.030 (methionine loss + acetylation), static cysteine modification of +57.0215 Da (carbamidomethylation), and static peptide N-terminal and lysine modifications of +304.2071 Da (TMTpro 16plex). Multiconsensus searching was used to combine time-resolved interactomics data from 8 replicates and 4 MS runs for SARS-CoV-2 nsp15 and data from 6 replicates and 3 MS runs for SARS-CoV nsp15.

The mass spectrometry proteomics data have been deposited to the ProteomeXchange Consortium via the PRIDE^58^ partner repository with the data set identifiers PXD063935 and 10.6019/PXD063935.

### 4.11 Mass Spectrometry Data Analysis

Raw TMT abundances from Proteome Discoverer were median-normalized and log_2_-transformed. The expression change between the continuous sample and the DMSO sample was calculated for each protein in each replicate and tested for significance with a paired *t*-test. Proteins with a log_2_(fold change) and p-value greater than 0.3 for SARS-CoV-2 and greater than 0.7 for SARS-CoV were considered to be interactors. The same comparison was made to the TMP control sample, and proteins with a log_2_(FC) and p-value greater than 0.5 for SARS-CoV-2 were added to the list of interactors. Only DMSO interactors were considered for SARS-CoV nsp15. Raw TMT abundances for the interactors in the time course samples were normalized to the bait and the DMSO sample.

## Supplementary Material























## Data Availability

Mass spectrometry data associated with our manuscript have been deposited to ProteomeXchange Consortium via the PRIDE partner repository with the data set identifier PXD063935.

## Notes

List of primers used for site-directed mutagenesis and construct design (XLSX)
Protein-level mass spectrometry data for SARS-CoV-2 nsp15 TRAPPED from Proteome Discoverer, 8 replicates (XLSX)
Peptide-level mass spectrometry data for SARS-CoV-2 nsp15 TRAPPED from Proteome Discoverer, 8 replicates (XLSX)
SubcellulaRVis for interactors of SARS-CoV-2 nsp15 (XLSX)
Reactome 2022 searched through EnrichR for SARS-CoV-2 nsp15 interactors (XLSX)
Mean and SEM of log_2_(FC) data for SARS-CoV-2 nsp15 interactors used for heatmap and individual traces (XLSX)
Protein-level mass spectrometry data for SARS-CoV nsp15 TRAPPED from Proteome Discoverer, 6 replicates (XLSX)
Peptide-level mass spectrometry data for SARS-CoV nsp15 TRAPPED from Proteome Discoverer, 6 replicates (XLSX)
SubcellulaRVis for interactors for SARS-CoV nsp15 (XLSX)
Mean of log_2_(FC) data for SARS-CoV nsp15 interactors used for heatmap and individual traces (XLSX)
Detailed synthetic scheme for compounds including detailed synthetic methods, characterization of the nonoptimal TRAP-cysteine pair, complete Western blot panels for affinity enrichment, characterization of mass spectrometry data quality, and ^1^H NMR, ^13^C NMR, LC–MS analysis of synthesized compounds (PDF)

## ⊥

R.M.C. College of Medicine, University of Illinois at Chicago, Chicago, IL, 60607, USA

## #

Lombardi Comprehensive Cancer Center, Georgetown University Medical Center, Washington, DC, 20007, USA.

## References

[1] Singh N., Bhalla N. (2020). Moonlighting Proteins. Annu.
Rev. Genet..

[2] Gómez A., Hernández S., Amela I., Piñol J., Cedano J., Querol E. (2011). Do Protein–Protein
Interaction
Databases Identify Moonlighting Proteins?. Mol.
BioSyst..

[3] Espinosa-Cantú A., Cruz-Bonilla E., Noda-Garcia L., DeLuna A. (2020). Multiple Forms of Multifunctional
Proteins in Health and Disease. Front Cell Dev
Biol..

[4] Ho J. S. Y., Zhu Z., Marazzi I. (2021). Unconventional Viral
Gene Expression
Mechanisms as Therapeutic Targets. Nature.

[5] Almasy K. M., Davies J. P., Plate L. (2021). Comparative
Host Interactomes of
the SARS-CoV-2 Nonstructural Protein 3 and Human Coronavirus Homologs. Mol Cell Proteomics.

[6] Gordon D. E., Jang G. M., Bouhaddou M., Xu J., Obernier K., White K. M., O’Meara M. J., Rezelj V. V., Guo J. Z., Swaney D. L. (2020). A SARS-CoV-2
Protein Interaction Map Reveals
Targets for Drug Repurposing. Nature.

[7] Gordon D. E., Hiatt J., Bouhaddou M., Rezelj V. V., Ulferts S., Braberg H., Jureka A. S., Obernier K., Guo J. Z., Batra J. (2020). Comparative
Host-Coronavirus Protein Interaction Networks
Reveal Pan-Viral Disease Mechanisms. Science.

[8] St-Germain J. R., Astori A., Samavarchi-Tehrani P., Abdouni H., Macwan V., Kim D.-K., Knapp J. J., Roth F. P., Gingras A.-C., Raught B. (2020). A SARS-CoV-2 BioID-Based
Virus-Host Membrane Protein
Interactome and Virus Peptide Compendium: New Proteomics Resources
for COVID-19 Research. bioRxiv.

[9] Samavarchi-Tehrani P., Abdouni H., Knight J. D. R., Astori A., Samson R., Lin Z.-Y., Kim D.-K., Knapp J. J., St-Germain J., Go C. D. (2020). A SARS-CoV-2—Host Proximity Interactome. bioRxiv.

[10] Pillon M. C., Frazier M. N., Dillard L. B., Williams J. G., Kocaman S., Krahn J. M., Perera L., Hayne C. K., Gordon J., Stewart Z. D. (2021). Cryo-EM
Structures of the SARS-CoV-2 Endoribonuclease
Nsp15 Reveal Insight into Nuclease Specificity and Dynamics. Nat. Commun..

[11] Hackbart M., Deng X., Baker S. C. (2020). Coronavirus
Endoribonuclease Targets
Viral Polyuridine Sequences to Evade Activating Host Sensors. Proc. Natl. Acad. Sci. U.S.A..

[12] Roth-Cross J. K., Bender S. J., Weiss S. R. (2008). Murine Coronavirus
Mouse Hepatitis
Virus Is Recognized by MDA5 and Induces Type I Interferon in Brain
Macrophages/Microglia. J. Virol..

[13] Yuen C.-K., Lam J.-Y., Wong W.-M., Mak L.-F., Wang X., Chu H., Cai J.-P., Jin D.-Y., To K. K.-W., Chan J. F.-W. (2020). SARS-CoV-2 Nsp13, Nsp14, Nsp15 and Orf6 Function as
Potent Interferon Antagonists. Emerging Microbes
& Infections.

[14] Li X., Cheng Z., Wang F., Chang J., Zhao Q., Zhou H., Liu C., Ruan J., Duan G., Gao S. (2021). A Negative Feedback Model to Explain Regulation of SARS-CoV-2 Replication
and Transcription. Front. Genet..

[15] Laurent E. M. N., Sofianatos Y., Komarova A., Gimeno J.-P., Tehrani P. S., Kim D.-K., Abdouni H., Duhamel M., Cassonnet P., Knapp J. J. (2020). Global BioID-Based SARS-CoV-2
Proteins Proximal
Interactome Unveils Novel Ties between Viral Polypeptides and Host
Factors Involved in Multiple COVID19-Associated Mechanisms. bioRxiv.

[16] Liu X., Huuskonen S., Laitinen T., Redchuk T., Bogacheva M., Salokas K., Pöhner I., Öhman T., Tonduru A. K., Hassinen A. (2021). SARS-CoV-2–Host
Proteome Interactions for Antiviral Drug Discovery. Molecular Systems Biology.

[17] May D. G., Martin-Sancho L., Anschau V., Liu S., Chrisopulos R. J., Scott K. L., Halfmann C. T., Díaz Peña R., Pratt D., Campos A. R. (2022). A BioID-Derived Proximity
Interactome for SARS-CoV-2 Proteins. Viruses.

[18] Meyers J. M., Ramanathan M., Shanderson R. L., Beck A., Donohue L., Ferguson I., Guo M. G., Rao D. S., Miao W., Reynolds D. (2021). The Proximal Proteome of 17 SARS-CoV-2 Proteins
Links to Disrupted Antiviral Signaling and Host Translation. PLOS Pathogens.

[19] Sharma A., Ong J. W., Loke M. F., Chua E. G., Lee J. J., Choi H. W., Tan Y. J., Lal S. K., Chow V. T. (2021). Comparative
Transcriptomic and Molecular Pathway Analyses of HL-CZ Human Pro-Monocytic
Cells Expressing SARS-CoV-2 Spike S1, S2, NP, NSP15 and NSP16 Genes. Microorganisms.

[20] Morris J. H., Knudsen G. M., Verschueren E., Johnson J. R., Cimermancic P., Greninger A. L., Pico A. R. (2014). Affinity purification–mass
spectrometry and network analysis to understand protein-protein interactions. Nat. Protoc..

[21] Dickson K. M., Bergeron J. J. M., Shames I., Colby J., Nguyen D. T., Chevet E., Thomas D. Y., Snipes G. J. (2002). Association of Calnexin
with Mutant Peripheral Myelin Protein-22 Ex Vivo: A Basis for “Gain-of-Function”
ER Diseases. Proc. Natl. Acad. Sci. U.S.A..

[22] Roux K. J., Kim D. I., Raida M., Burke B. (2012). A promiscuous biotin
ligase fusion protein identifies proximal and interacting proteins
in mammalian cells. J. Cell Biol..

[23] Susa K. J., Bradshaw G. A., Eisert R. J., Schilling C. M., Kalocsay M., Blacklow S. C., Kruse A. C. (2024). A Spatiotemporal
Map of Co-Receptor Signaling Networks Underlying B Cell Activation. Cell Reports.

[24] Zhong X., Li Q., Polacco B. J., Patil T., Marley A., Foussard H., Khare P., Vartak R., Xu J., DiBerto J. F. (2024). A Proximity
Proteomics Pipeline with Improved Reproducibility and
Throughput. Molecular Systems Biology.

[25] Polacco B. J., Lobingier B. T., Blythe E. E., Abreu N., Khare P., Howard M. K., Gonzalez-Hernandez A.
J., Xu J., Li Q., Novy B. (2024). Profiling the Proximal Proteome of the Activated
μ-Opioid Receptor. Nat. Chem. Biol..

[26] Martin A. P., Bradshaw G. A., Eisert R. J., Egan E. D., Tveriakhina L., Rogers J. M., Dates A. N., Scanavachi G., Aster J. C., Kirchhausen T. (2023). A Spatiotemporal Notch
Interaction Map from Plasma Membrane to Nucleus. Sci. Signal.

[27] Ong S.-E., Blagoev B., Kratchmarova I., Kristensen D. B., Steen H., Pandey A., Mann M. (2002). Stable Isotope Labeling
by Amino Acids in Cell Culture, SILAC, as a Simple and Accurate Approach
to Expression Proteomics*. Molecular & Cellular
Proteomics.

[28] Noren C. J., Anthony-Cahill S. J., Griffith M. C., Schultz P. G. (1989). A General Method
for Site-Specific Incorporation of Unnatural Amino Acids into Proteins. Science.

[29] Wright M. T., Timalsina B., Garcia Lopez V., Hermanson J. N., Garcia S., Plate L. (2024). Time-resolved interactome profiling
deconvolutes secretory protein quality control dynamics. Molecular Systems Biology.

[30] Sakamoto K. M., Kim K. B., Kumagai A., Mercurio F., Crews C. M., Deshaies R. J. (2001). Protacs: Chimeric Molecules That
Target Proteins to
the Skp1–Cullin–F Box Complex for Ubiquitination and
Degradation. Proc. Natl. Acad. Sci. U.S.A..

[31] Iwamoto M., Björklund T., Lundberg C., Kirik D., Wandless T. J. (2010). A General
Chemical Method to Regulate Protein Stability in the Mammalian Central
Nervous System. Chem. Biol..

[32] Miyazaki Y., Imoto H., Chen L., Wandless T. J. (2012). Destabilizing Domains
Derived from the Human Estrogen Receptor. J.
Am. Chem. Soc..

[33] Banaszynski L. A., Chen L., Maynard-Smith L. A., Ooi A. G. L., Wandless T. J. (2006). A Rapid,
Reversible, and Tunable Method to Regulate Protein Function in Living
Cells Using Synthetic Small Molecules. Cell.

[34] Navarro R., Chen L., Rakhit R., Wandless T. J. (2016). A Novel Destabilizing
Domain Based on a Small-Molecule Dependent Fluorophore. ACS Chem. Biol..

[35] Shoulders M. D., Ryno L. M., Genereux J. C., Moresco J. J., Tu P. G., Wu C., Yates J. R., Su A. I., Kelly J. W., Wiseman R. L. (2013). Stress-Independent
Activation of XBP1s and/or ATF6 Reveals Three Functionally Diverse
ER Proteostasis Environments. Cell Reports.

[36] Giadone R. M., Liberti D. C., Matte T. M., Rosarda J. D., Torres-Arancivia C., Ghosh S., Diedrich J. K., Pankow S., Skvir N., Jean J. C. (2020). Expression
of Amyloidogenic Transthyretin Drives Hepatic
Proteostasis Remodeling in an Induced Pluripotent Stem Cell Model
of Systemic Amyloid Disease. Stem Cell Reports.

[37] Matthews D. A., Bolin J. T., Burridge J. M., Filman D. J., Volz K. W., Kraut J. (1985). Dihydrofolate Reductase.
The Stereochemistry of Inhibitor Selectivity. J. Biol. Chem..

[38] Jing C., Cornish V. W. (2013). A Fluorogenic TMP-Tag for High Signal-to-Background
Intracellular Live Cell Imaging. ACS Chem. Biol..

[39] Polshakov V. I., Smirnov E. G., Birdsall B., Kelly G., Feeney J. (2002). Letter to
the Editor: NMR-Based Solution Structure of the Complex of Lactobacillus
Casei Dihydrofolate Reductase with Trimethoprim and NADPH. J. Biomol. NMR.

[40] Manna M. S., Tamer Y. T., Gaszek I., Poulides N., Ahmed A., Wang X., Toprak F. C. R., Woodard D. R., Koh A. Y., Williams N. S. (2021). A Trimethoprim Derivative Impedes Antibiotic
Resistance Evolution. Nat. Commun..

[41] Chu Z., Wang C., Tang Q., Shi X., Gao X., Ma J., Lu K., Han Q., Jia Y., Wang X. (2018). Newcastle Disease Virus V Protein Inhibits Cell Apoptosis and Promotes
Viral Replication by Targeting CacyBP/SIP. Front.
Cell. Infect. Microbiol..

[42] Meertens L., Hafirassou M. L., Couderc T., Bonnet-Madin L., Kril V., Kümmerer B. M., Labeau A., Brugier A., Simon-Loriere E., Burlaud-Gaillard J. (2019). FHL1 Is a Major Host
Factor for Chikungunya Virus Infection. Nature.

[43] Chen E. Y., Tan C. M., Kou Y., Duan Q., Wang Z., Meirelles G. V., Clark N. R., Ma’ayan A. (2013). Enrichr: interactive
and collaborative HTML5 gene list enrichment analysis tool. BMC Bioinf..

[44] Watson J., Smith M., Francavilla C., Schwartz J.-M. (2022). SubcellulaRVis:
A Web-Based Tool to Simplify and Visualise Subcellular Compartment
Enrichment. Nucleic Acids Res..

[45] Hayn M., Hirschenberger M., Koepke L., Nchioua R., Straub J. H., Klute S., Hunszinger V., Zech F., Prelli Bozzo C., Aftab W. (2021). Systematic Functional Analysis of SARS-CoV-2 Proteins
Uncovers Viral Innate Immune Antagonists and Remaining Vulnerabilities. Cell Reports.

[46] Topolska-Woś A.
M., Chazin W. J., Filipek A. (2016). CacyBP/SIP — Structure and
Variety of Functions. Biochimica et Biophysica
Acta (BBA) - General Subjects.

[47] Kilanczyk E., Filipek S., Filipek A. (2011). ERK1/2 Is Dephosphorylated
by a Novel
Phosphatase—CacyBP/SIP. Biochem. Biophys.
Res. Commun..

[48] Engler M., Albers D., Von Maltitz P., Groß R., Münch J., Cirstea I. C. (2023). ACE2-EGFR-MAPK Signaling
Contributes
to SARS-CoV-2 Infection. Life Science Alliance.

[49] Sun L., Chen L., Zhu H., Li Y., Chen C. C., Li M. (2021). FHL1 Promotes Glioblastoma Aggressiveness through Regulating EGFR
Expression. FEBS Lett..

[50] Sheikh F., Raskin A., Chu P.-H., Lange S., Domenighetti A. A., Zheng M., Liang X., Zhang T., Yajima T., Gu Y. (2008). An FHL1-Containing Complex
within the Cardiomyocyte
Sarcomere Mediates Hypertrophic Biomechanical Stress Responses in
Mice. J. Clin. Invest..

[51] Garcia-Moreno M., Noerenberg M., Ni S., Järvelin A. I., González-Almela E., Lenz C. E., Bach-Pages M., Cox V., Avolio R., Davis T. (2019). System-Wide Profiling
of RNA-Binding Proteins Uncovers Key Regulators of Virus Infection. Mol. Cell.

[52] Kamel W., Noerenberg M., Cerikan B., Chen H., Järvelin A. I., Kammoun M., Lee J. Y., Shuai N., Garcia-Moreno M., Andrejeva A. (2021). Global Analysis of Protein-RNA
Interactions
in SARS-CoV-2-Infected Cells Reveals Key Regulators of Infection. Mol. Cell.

[53] Cooke A., Schwarzl T., Huppertz I., Kramer G., Mantas P., Alleaume A.-M., Huber W., Krijgsveld J., Hentze M. W. (2019). The RNA-Binding Protein YBX3 Controls Amino Acid Levels
by Regulating SLC mRNA Abundance. Cell Reports.

[54] Zhao H., Cai Z., Rao J., Wu D., Ji L., Ye R., Wang D., Chen J., Cao C., Hu N. (2024). SARS-CoV-2 RNA Stabilizes Host mRNAs to Elicit
Immunopathogenesis. Mol. Cell.

[55] Ramadurgum P., Hulleman J. D. (2020). Protocol for Designing
Small-Molecule-Regulated Destabilizing
Domains for In Vitro Use. STAR Protocols.

[56] Nakahara E., Mullapudi V., Collier G. E., Joachimiak L. A., Hulleman J. D. (2022). Development of a
New DHFR-Based Destabilizing Domain
with Enhanced Basal Turnover and Applicability in Mammalian Systems. ACS Chem. Biol..

[57] Fonslow B. R., Niessen S. M., Singh M., Wong C. C. L., Xu T., Carvalho P. C., Choi J., Park S. K., Yates J. R. I. (2012). Single-Step
Inline Hydroxyapatite Enrichment Facilitates Identification and Quantitation
of Phosphopeptides from Mass-Limited Proteomes with MudPIT. J. Proteome Res..

[58] Perez-Riverol Y., Bandla C., Kundu D. J., Kamatchinathan S., Bai J., Hewapathirana S., John N. S., Prakash A., Walzer M., Wang S. (2025). The PRIDE Database at
20 Years: 2025 Update. Nucleic Acids Res..

