# Supplementary material for: Time-Resolved Analysis of Protein–Protein Ensembles Using a Destabilizing Domain to Map Dynamic Interactions of SARS-CoV‑2 nsp15
Source: ACS Chem Biol. 2025 Sep 1;20(9):2229–42. doi: 10.1021/acschembio.5c00377 (PMC12455562; doi:10.1021/acschembio.5c00377)
Supplement: Supplementary file 11 [file cb5c00377_si_011.pdf]

SUPPLEMENTAL INFORMATION

for

Time-Resolved Analysis of Protein-Protein Ensembles using a Destabilizing Domain (TRAPPED) to map dynamic interactions of SARS-CoV-2 nsp15

Crissey Cameron[1], R. Mason Clark[2#], Adam M. Metts[1], Runze M. Jiang[1], Toya D. Scaggs[3], Kwangho Kim[1,3], Gary A. Sulikowski[1,3], Lars Plate[1,2,4*]

[1]Department of Chemistry, Vanderbilt University, Nashville, TN, 37235, USA

[2]Department of Biological Sciences, Vanderbilt University, Nashville, TN, 37235, USA
[3]Vanderbilt Institute of Chemical Biology, Molecular Design and Synthesis Center, Vanderbilt University, Nashville, TN, 37235, USA

[4]Department of Pathology, Microbiology and Immunology, Vanderbilt University Medical Center, Nashville, TN, 37235, USA

Present address:

[5]R.M.C. College of Medicine, University of Illinois at Chicago, Chicago, IL, 60607, USA

[6]Lombardi Comprehensive Cancer Center, Georgetown University Medical Center, Washington, DC, 20007, USA

*Correspondence: lars.plate@vanderbilt.edu

Supplemental Information

Figure S1. Synthetic scheme for compounds **4**, **5**, and **6** for synthesis in 4-5 steps 3
Figure S2. In vitro analysis of reaction time between ecDHFR^{P55C}-YFP and vinyl ketone TRAP **6** 8
Figure S3. Quantification of Western blot abundance of stabilized DHFR(DD)^{L28C}-YFP and -SARS-CoV-2 nsp15 constructs 9
Figure S4. Western blot analysis of stabilization and labeling during affinity enrichment 10
Figure S5. Mass spectrometry characterization of SARS-CoV-2 nsp15 TRAPPED 11
Figure S6. Mass spectrometry characterization of SARS-CoV nsp15 TRAPPED 13
Figure S7. ^{1}H NMR of Compound 1 in DMSO-d6 14
Figure S8. ^{13}C NMR of Compound 1 in DMSO-d6 15
Figure S9. LC/MS of Compound **1** (expected MW: 277.1) 16
Figure S10. ^{1}H NMR of Compound **2** in MeOD 17
Figure S11. ^{13}C NMR of Compound **2** in MeOD 18
Figure S12. LC/MS of Compound **2** (expected MW: 334.2) 19
Figure S13. ^{1}H NMR of Compound **3** in MeOD 20
Figure S14. ^{13}C NMR of Compound **3** in MeOD 21
Figure S15. LC/MS of Compound **3** (expected MW: 429.2) 22
Figure S16. ^{1}H NMR of Compound **3a** in MeOD 23
Figure S17. ^{13}C NMR of Compound **3a** in MeOD 24
Figure S18. ^{1}H NMR of Compound **4** in MeOD 25
Figure S19. HRMS of Compound **4** (expected spectra shown) 26
Figure S20. ^{1}H NMR of Compound **5** in MeOD 27
Figure S21. HRMS of Compound **5** (expected spectra shown) 28
Figure S22. ^{1}H NMR of Compound **6** in MeOD 29
Figure S23. ^{13}C NMR of Compound **6** in MeOD 30
Figure S24. HRMS of Compound **6** (expected spectra shown) 31

A

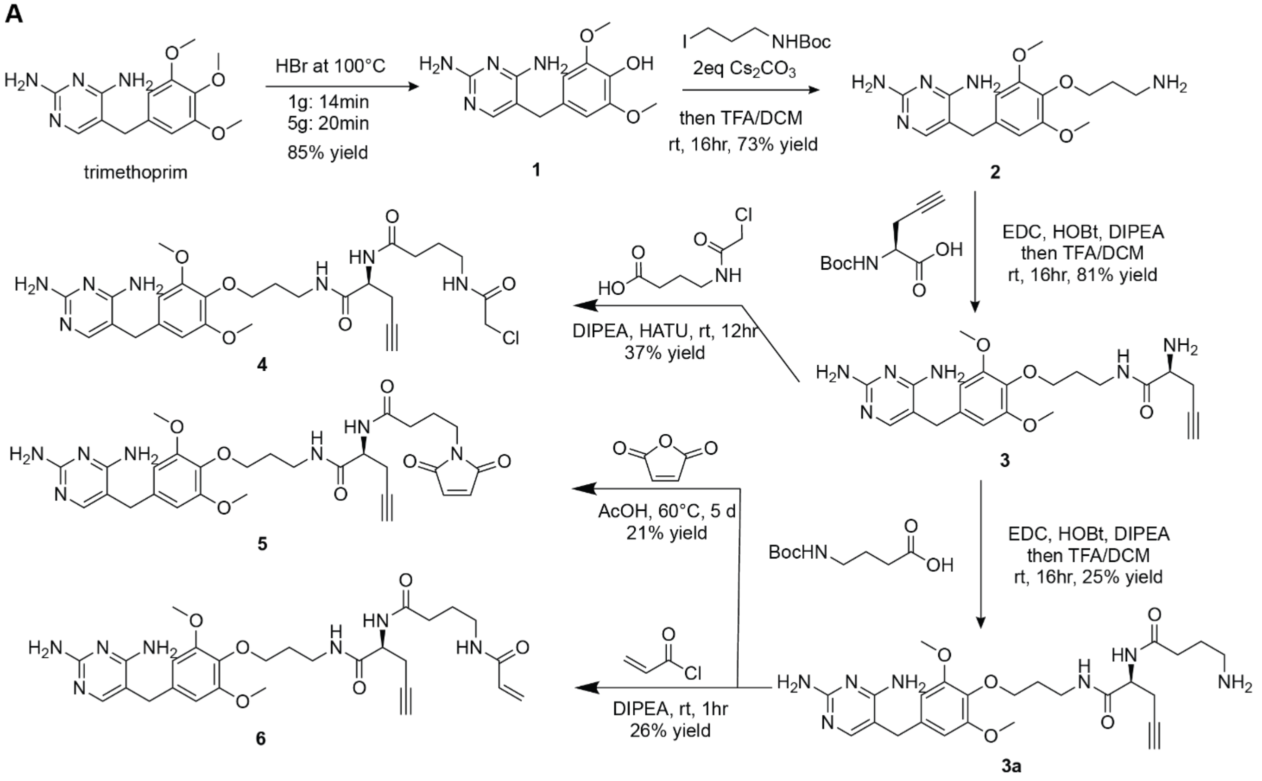

Figure S1. Synthetic scheme for compounds 4, 5, and 6 for synthesis in 4-5 steps. (A) Detailed synthetic scheme for compounds **4**, **5**, and **6**.

Synthesis of compound **1**: 4-((2,4-diaminopyrimidin-5-yl)methyl)-2,6-dimethoxyphenol:
In a 200 mL round bottom flask equipped with a magnetic stir bar, aqueous 48% hydrobromic acid (60 mL, 0.53 mol) is allowed to warm in a 100°C oil bath. Trimethoprim (5 g, 17.2 mmol) is added to the flask once heated and a Liebig condenser is attached. The solution is stirred for exactly 20 minutes before ice-cold aqueous 25% sodium hydroxide (12 mL, 75 mmol) is added down the condenser to quench the reaction. The timing is crucial to optimize the ratio of product to unreacted trimethoprim and doubly demethylated trimethoprim. The mixture is cooled overnight at 4°C and filtered the next morning. The crystals are collected and redissolved in a minimal amount of boiling distilled water before being neutralized with concentrated ammonium hydroxide solution to a pH of 7. The mixture is again allowed to recrystallize overnight at 4°C before filtering and collecting the product as 4.08 g (85% yield) tan crystals. This procedure was also performed on a 1 g scale with the same ratio of reagents, but the trimethoprim is only allowed to stir in the acid for 14 minutes before quenching. ^{1}H NMR (**Figure S7**, 400 MHz, DMSO-d6) δ ppm: 7.45 (s, 1H), 6.48 (s, 2H), 3.69 (s, 6H), 3.47 (s, 2H). ^{13}C NMR (**Figure S8**, 600 MHz, DMSO-d6) δ ppm: 163.88,

157.10, 148.43, 134.48, 128.49, 108.97, 106.83, 56.50, 32.67. Compound **1** m/z calcd. for $C_{13}H_{16}N_4O_3$: 277.1. Found: 277.3. (**Figure S9**)

Synthesis of tert-butyl (3-iodopropyl)carbamate for synthesis of **2**:
In a 200 mL round bottom flask, a stirring solution of triphenylphosphane (1.50 g, 5.71 mmol) and 1H-imidazole (388.5 mg, 5.71 mmol) under argon in dichloromethane (DCM) (40 mL) was mixed with diiodine (1.74 g, 6.85 mmol) at 0°C. Upon iodine addition, the solution went from clear to a bright yellow, and subsequently a dark red. The reaction mixture was stirred for 5 minutes before tert-butyl (3-hydroxypropyl)carbamate (1.00 g, 5.71 mmol) in DCM (6 mL) was added dropwise. The reaction was allowed to warm to room temperature and stir for 3.5 hours before the reaction mixture was washed successively with water (3 times), saturated sodium thiosulfate solution (2 times), and brine (2 times). The organic layer was then dried over anhydrous magnesium sulfate, filtered, and concentrated in vacuo. The crude, yellow product was then subjected to column chromatography (30% EtOAc in Hexanes) to yield 1.187 g of the above-named product as a yellow oil (73.0% yield).

Synthesis of compound **2**: 5-(4-(3-aminopropoxy)-3,5-dimethoxybenzyl)pyrimidine-2,4-diamine
In a 200 mL round bottom flask equipped with a magnetic stirring bar, compound **4** (1.15 g, 4.2 mmol, 1 equiv), tert-butyl (3-iodopropyl)carbamate (1.19 g, 4.2 mmol, 1 equiv), and cesium carbonate (2.71 g, 8.4 mmol, 2 equiv) were dissolved in 12 mL dimethylformamide (DMF) and stirred at 70° C for 12 hours. The reaction mixture was concentrated in vacuo and then subjected to column chromatography (7% 1:9 NH_4OH:MeOH in DCM) to yield the Boc-protected product as a yellow oil. The product was dissolved in 10 mL of 50% trifluoroacetic acid (TFA) in DCM and stirred at room temperature until complete deprotection was observed by thin layer chromatography (TLC), approximately 30 minutes, yielding the title compound as the trifluoroacetate salt as a white solid (868 mg, 67% yield). ^{1}H NMR (**Figure S10**, 400 MHz, MeOD) δ ppm: 7.26 (s, 1H), 6.62 (s, 2H), 4.11 (t, J = 5.4 Hz, 2H), 3.85 (s, 6H), 3.69 (s, 2H), 3.26 (t, J = 6.2 Hz, 2 H), 2.06 (m, 2 H). ^{13}C NMR (**Figure S11**, 600 MHz, MeOD) δ ppm: 166.11, 156.28, 154.48, 140.46, 136.39, 134.36, 110.69, 106.97, 73.44, 56.55, 40.32, 33.93, 28.28. Compound **2** m/z calcd. for $C_{16}H_{23}N_5O_3$: 334.2. Found: 334.3. (**Figure S12**)

Synthesis of compound **3**: (S)-2-amino-N-(3-(4-((2,4-diaminopyrimidin-5-yl)methyl)-2,6-dimethoxyphenoxy)propyl)pent-4-ynamide

In a 200 mL round bottom flask equipped with a magnetic stirring bar, compound **5** (361 mg, 1.08 mmol, 1 equiv) and diisopropylethylamine (0.94 mL, 5.41 mmol, 5 equiv) were mixed under argon gas and stirred at room temperature for 15 minutes. Hydroxybenzotriazole hydrate (41.5 mg, 0.27 mmol, 0.25 equiv) and (S)-2-((tert-butoxycarbonyl)amino)pent-4-ynoic acid (254 mg, 1.19 mmol, 1.1 equiv) were mixed in a separate vessel for 5 minutes and then added to the main flask and stirred for 15 minutes. 1-Ethyl-3-(3-dimethylaminopropyl)carbodiimide (EDC) (623 mg, 3.25 mmol, 3 equiv) was added to the flask and the final mixture was allowed to stir for 12 hours. The reaction mixture was concentrated in vacuo and subjected to column chromatography (7% 1:9 NH_4OH:MeOH in DCM) to yield the Boc-protected product as a yellow oil. The product was dissolved in 10 mL of 50% TFA in DCM and stirred at room temperature until complete deprotection was observed by TLC, approximately 30 minutes, yielding the title compound as the trifluoroacetate salt as a white solid (269 mg, 58% yield). ^{1}H NMR (**Figure S13**, 400 MHz, MeOD) δ ppm: 7.25 (s, 1H), 6.57 (s, 2H), 3.99 (m, 3H), 3.82 (s, 6H), 3.67 (s, 2H), 3.54 (m, 1H), 3.47 (m, 1H) 2.82 (m, 2H), 2.59 (m, 1H), 1.92 (m, 2H). ^{13}C NMR (**Figure S14**, 600 MHz, MeOD) δ ppm: 168.56,166.09, 156.22, 154.88, 140.43, 136.78, 133.89, 110.74, 107.11, 77.32, 74.79, 72.14, 57.58, 52.89, 38.35, 33.88, 30.59, 22.32. Compound **3** m/z calcd. for $C_{21}H_{28}N_6O_4$: 429.2. Found: 429.4. (**Figure S15**)

Synthesis of compound **3a**: (S)-2-(4-aminobutanamido)-N-(3-(4-((2,4-diaminopyrimidin-5-yl)methyl)-2,6-dimethoxyphenoxy)propyl)pent-4-ynamide

In a 100 mL round bottom flask equipped with a magnetic stirring bar, compound **3** (369 mg, 0.86 mmol, 1 equiv) and diisopropylethylamine (0.75 mL, 4.31 mmol, 5 equiv) were mixed under argon gas and stirred at room temperature for 15 minutes. Hydroxybenzotriazole hydrate (33 mg, 0.22 mmol, 0.25 equiv) and 4-((tert-butoxycarbonyl)amino)butanoic acid (192.5 mg, 0.95 mmol, 1.1 equiv) were mixed in a separate vessel for 5 minutes and then added to the main flask and stirred for 15 minutes. EDC (495 mg, 2.58 mmol, 3 equiv) was added to the flask and the final mixture was allowed to stir for 12 hours. The reaction mixture was concentrated in vacuo and subjected to column chromatography (7% 1:9 NH_4OH:MeOH in DCM) to yield the Boc-protected product as a yellow oil. The product was dissolved in 10 mL of 50% TFA in DCM and stirred at room

temperature until complete deprotection was observed by TLC, around 30 minutes, yielding the title compound as the trifluoroacetate salt as a white solid (82.5 mg, 19% yield). ^{1}H NMR (**Figure S16**, 400 MHz, MeOD) δ ppm: 7.26 (s, 1H), 6.58 (s, 2H), 4.46 (dd, 1H), 3.98 (dd, 2H), 3.82 (s, 6H), 3.67 (s, 2H), 3.47 (m, 2H), 2.96 (t, 2H), 2.67 (m, 2H), 2.35 (m, 3H), 1.90 (sep, 4), 1.36 (m, 1H). ^{13}C NMR (**Figure S17**, 600 MHz, MeOD) δ ppm: 173.10, 170.84, 170.75, 164.76, 154.79, 153.51, 139.00, 135.49, 132.55, 109.40, 105.74, 78.93, 71.17, 70.92, 55.22, 52.47, 42.03, 38.81, 37.09, 36.96, 32.45, 31.89, 29.16, 22.78, 21.20.

Synthesis of compound **4**: (S)-2-(4-(2-chloroacetamido)butanamido)-N-(3-(4-((2,4-diaminopyrimidin-5-yl)methyl)-2,6-dimethoxyphenoxy)propyl)pent-4-ynamide

In an oven-dried microwave vial equipped with a magnetic stir bar, compound **3** (40 mg, 61 μmol, 1 equiv), 4-(2-chloroacetamido)butanoic acid (13 mg, 74 μmol, 1.2 equiv) were suspended in dry DMF (0.3 mL) under argon gas then stirred. Added DIPEA (5 equiv). Added solution of HATU (34 mg, 88 μmol, 1.2 equiv) in dry DMF (0.2 mL). The reaction was stirred at room temperature until consumption of starting material (typically 12 hours). The reaction was poured into H_2O then extracted with EtOAc (3 times). Combined organic layers were washed with H_2O (3 times), dried over anhydrous Na_2SO_4, filtered, and concentrated in vacuo. The crude residue was purified by flash chromatography (12 g silica cartridge; MeOH/DCM: product eluted at 30% MeOH/DCM) providing the title compound as an off-white solid (16 mg, 37% yield). ^{1}H NMR (**Figure S18**, 600 MHz, MeOD) δ ppm: 7.27 (m, 1H), 6.58 (s, 2H), 4.46 (dd, 1H), 4.03 (s, 2H), 3.98 (t, 2H), 3.82 (s, 6H), 3.67 (s, 2H), 3.45 (m, 2H), 3.22 (m, 4H), 3.05 (s, 1H), 2.67 (m, 2H), 2.35 (t, 1H), 2.23 (m, 2H), 1.89 (quint, 2H), 1.77 (m, 2H). Compound **4** m/z calcd. for $C_{27}H_{36}CN_7O_6$ [M+H]: 590.2488. Found: 590.2487. (**Figure S19**)

Synthesis of compound **5**: (S)-N-(3-(4-((2,4-diaminopyrimidin-5-yl)methyl)-2,6-dimethoxyphenoxy)propyl)-2-(4-(2,5-dioxo-2,5-dihydro-1H-pyrrol-1-yl)butanamido)pent-4-ynamide

In a dried microwave vial equipped with a magnetic stir bar, the amine TFA salt (120 mg, 0.16 mmol, 1 equiv) and maleic anhydride (64 mg, 0.65 mmol, 4 equiv) were suspended in glacial AcOH (2 mL). The reaction mixture was heated to 60 °C and stirred for 5 days. The reaction mixture was concentrated in vacuo. The crude residue was purified by flash chromatography (12

g silica cartridge; MeOH/DCM: product eluted at 30% MeOH/DCM) providing the title compound as an off-white solid (20 mg, 21% yield). ^{1}H NMR (**Figure S20**, 600 MHz, MeOD) δ ppm: 7.27 (s, 1H), 6.80 (s, 2H), 6.58 (s, 2H), 6.27 (s, 3H), 4.45 (dd, 1H), 3.98 (m, 2H), 3.81 (s, 6H), 3.67 (s, 2H), 3.45 (m, 4H), 3.24 (m, 1H), 2.67 (m, 2H), 2.34 (t, 1H), 2.20 (m, 2H), 1.89 (quint, 2H), 1.81 (m, 2H). Compound **5** m/z calcd. for $C_{29}H_{36}N_7O_7$ [M+H]: 594.2671. Found: 594.2668. (**Figure S21**)

Synthesis of compound **6**: (S)-2-(4-acrylamidobutanamido)-N-(3-(4-((2,4-diaminopyrimidin-5-yl)methyl)-2,6-dimethoxyphenoxy)propyl)pent-4-ynamide

In an oven-dried 10 mL round bottom flask equipped with a magnetic stir bar, compound **3a** (82.5 mg, 161 µmol, 1 equiv) was suspended in dry DMF (2mL) under Ar and cooled to 0 °C in an ice bath. Once cold, diisopropylethylamine (140 µL, 804 µmol, 5 equiv) was added dropwise. After stirring for 5 minutes, freshly distilled acryloyl chloride (20 µL, 241 µmol, 1.5 equiv) was 29 added by syringe. The stirring solution was warmed to room temperature and allowed to stir for 1 hour before concentrating the reaction mixture *in vacuo*. The crude residue was purified by flash chromatography (12 g silica cartridge; MeOH/DCM: product eluted at 30% MeOH/DCM) providing the title compound as an off-white solid (24 mg, 26% yield). ^{1}H NMR (**Figure S22**, 600 MHz, MeOD) δ ppm: 7.41 (s, 1H), 6.56 (s, 2H), 6.23 (m, 2H), 5.64 (m, 1H), 4.47 (dd, 1H), 3.97 (t, 2H), 3.80 (s, 6H), 3.72 (m, 2H), 3.66 (s, 2H), 3.45 (m, 2H), 3.22 (m, 3H), 2.67 (m, 2H), 2.37 (t, 1H), 2.25 (m, 2H), 1.88 (quint, 2H), 1.78 (quint, 2H). ^{13}C NMR (**Figure S23**, 600 MHz, MeOD) δ ppm: 175.42, 172.23, 168.11, 165.27, 159.57, 154.80, 148.06, 136.70, 135.25, 132.06, 126.66, 109.52, 106.95, 80.49, 72.71, 72.32, 56.66, 55.78, 53.87, 49.84, 43.78, 39.66, 38.43, 34.16, 33.84, 30.67, 30.55, 26.47, 26.40, 22.54, 18.02, 13.19. Compound **6** m/z calcd. for $C_{28}H_{38}N_7O_6$ [M+H]: 568.2878. Found: 568.2883. (**Figure S24**)

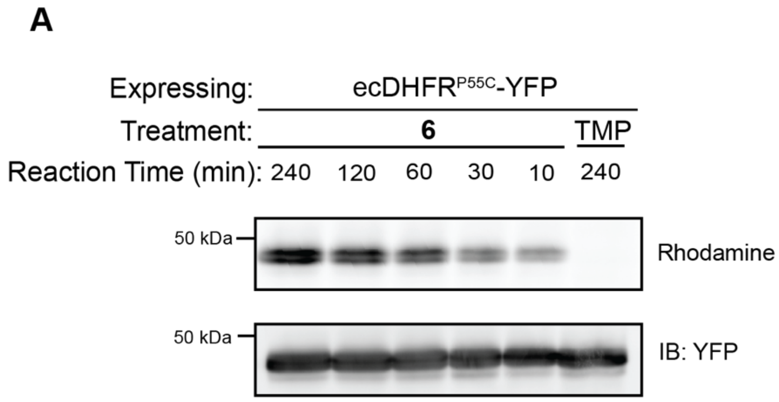

Figure S2. In vitro analysis of reaction time between ecDHFR^{P55C}-YFP and vinyl ketone TRAP 6: (A) Western blot of HEK293T lysates expressing ecDHFR^{P55C}-YFP incubated with 10 μM TRAP **6** for various time points before being quenched with 1mM β-mercaptoethanol and conjugated to TAMRA-Azide-PEG-Desthiobiotin.

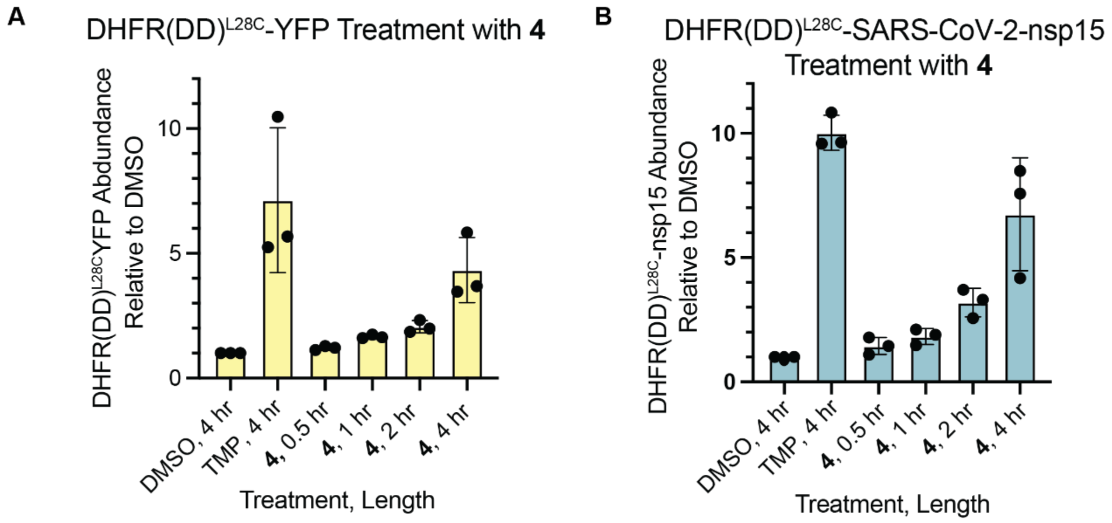

Figure S3. Quantification of Western blot abundance of stabilized DHFR(DD)^{L28C}-YFP and -SARS-CoV-2 nsp15 constructs: (A) Quantification of blots in Figure 3A, 3 replicates. DHFR(DD)^{L28C}-YFP abundance relative to DMSO treatment in Western blot analysis of HEK293T cells transfected with DHFR(DD)^{L28C}-YFP and treated with DMSO, 10 µM TMP, or 10 µM **4** for different durations of time. (B) Quantification of blots in Figure 3C, 3 replicates. DHFR(DD)^{L28C}-SARS-CoV-2-nsp15 abundance relative to DMSO treatment in Western blot analysis of HEK293T cells transfected with DHFR(DD)^{L28C}- SARS-CoV-2-nsp15 and treated with DMSO, 10 µM TMP, or 10 µM **4** for different durations of time.

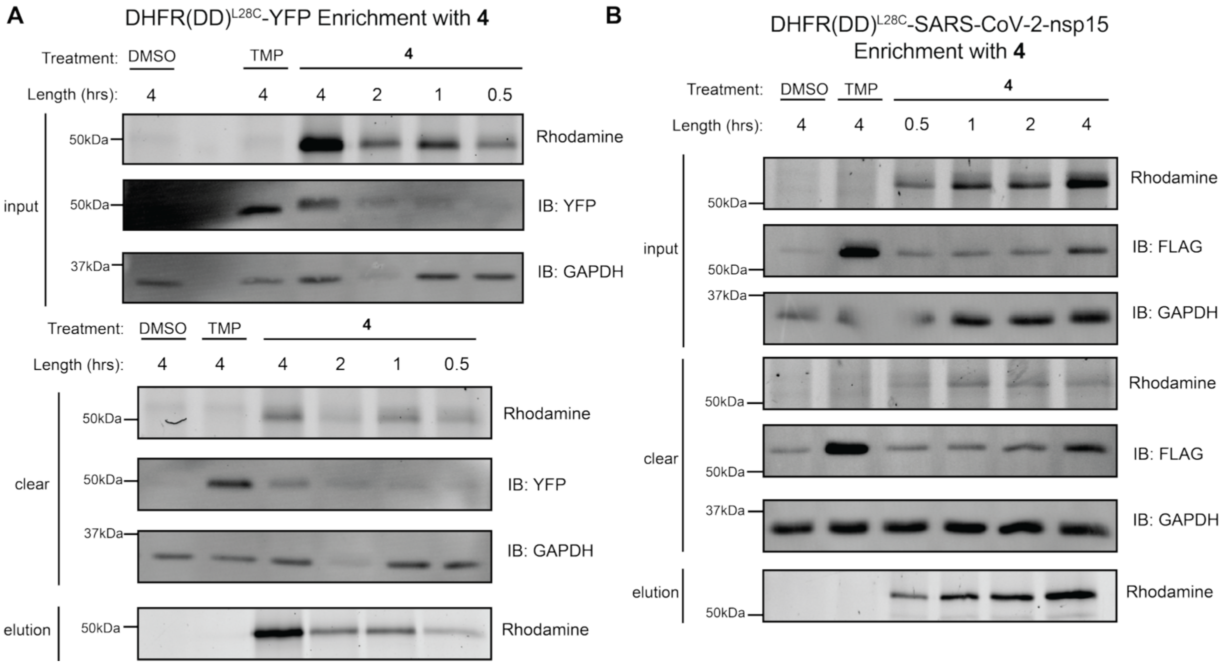

Figure S4. Western blot analysis of stabilization and labeling during affinity enrichment: (A) Images of rhodamine and YFP intensity using Western blot analysis of from DHFR(DD)^{L28C}-YFP transfected HEK293T cells treated with DMSO, TMP, or **4** after binding labeled sample to streptavidin beads (clear). Gel and blot images from the sample before incubation with the beads (input) and after elution from the beads (elution) are also shown. YFP labeled with **4** is clearly prominent in the input and elution images (rhodamine), but markedly more faint in the clear while YFP stabilized with TMP (FLAG) is present in input and clear images only and therefore is not enriched using the beads. (B) Images of rhodamine and YFP intensity using Western blot analysis of from DHFR(DD)^{L28C}-YFP transfected HEK293T cells treated with DMSO, TMP, or **4**, clear samples. Gel and blot images from the sample before incubation with the beads (input) and after elution from the beads (elution) are also shown. Signal from the rhodamine functionalization is clearly prominent in the input and elution images for samples labeled with TRAP **4**, but much less prominent in the clear image, indicating that most of the POI that was labeled by the TRAP is enriched. FLAG signal in the TMP-treated sample is only present in the input and the clear images, which supports that the POI population that is stabilized but not functionalized by the TRAP is unable to be enriched.

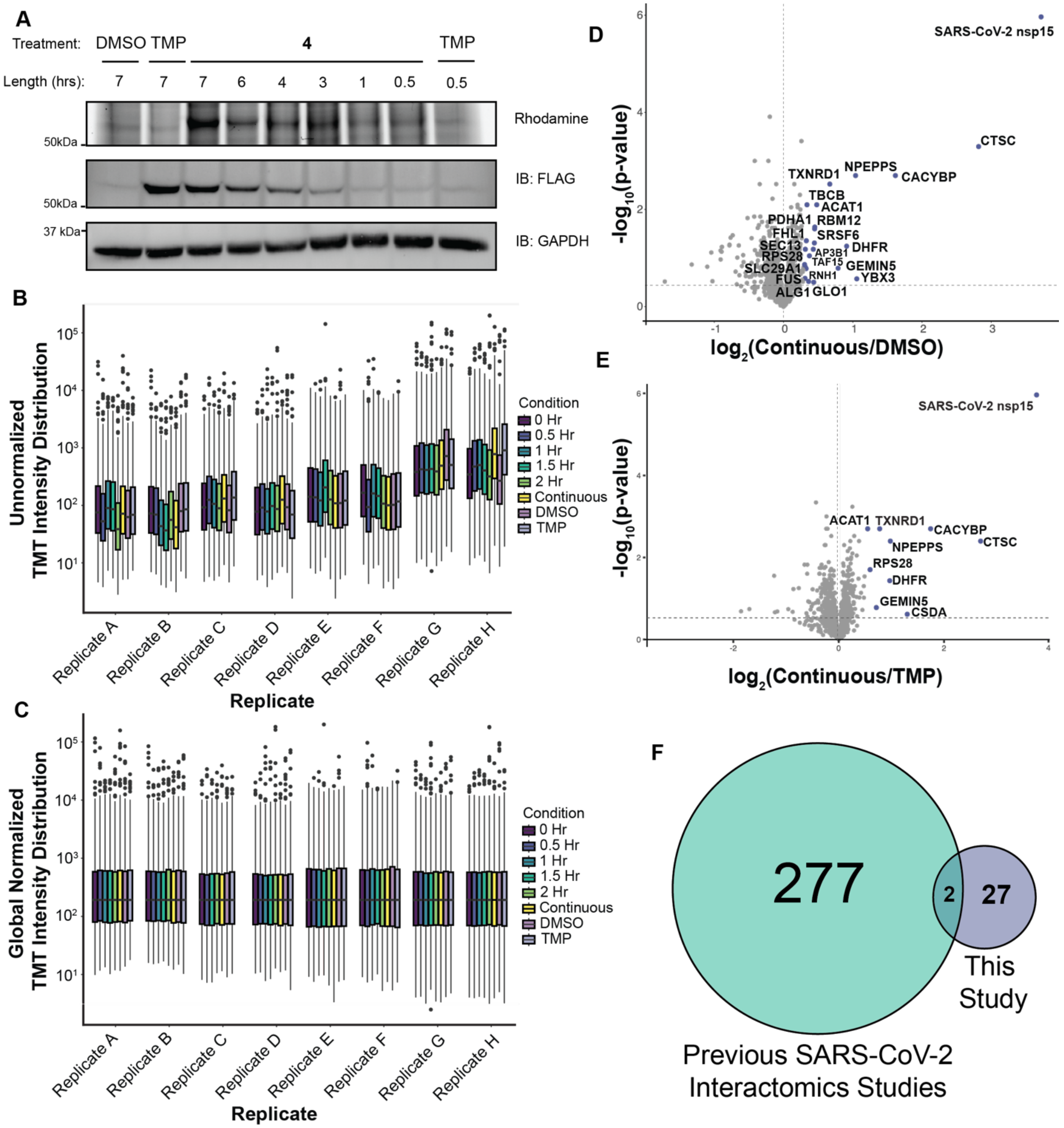

Figure S5. Mass spectrometry characterization of SARS-CoV-2 nsp15 TRAPPED: (A) Western blot analysis of HEK293T cells transfected with FT-DHFR(DD)^{L28C}-nsp15 and treated with DMSO, 10µM TMP, or 10 µM **4** for a range of time periods to determine the appropriate pulse length. 30 minutes was determined to produce a signal that was detectable above background signal observed in DMSO and TMP samples, and therefore, a 30-minute pulse period was chosen for TRAPPED experiments. (B) Raw distribution of TMT abundances for all proteins identified

in 8 replicates of pulse chase data analyzed by MS before normalization. (C) Distribution of TMT abundances for all proteins identified in 8 replicates of pulse chase data analyzed by MS after median normalization. (D) Volcano plot comparing the enrichment of nsp15 interactors in continuous sample to vehicle control. Interactors with $\log_2$(fold change) greater than 0.3 and -$\log_{10}$(p-value) greater than 0.3 are highlighted in purple and annotated. (E) Volcano plot comparing the enrichment of nsp15 interactors in continuous sample to TMP negative enrichment control. Interactors with $\log_2$(fold change) greater than 0.5 and -$\log_{10}$(p-value) greater than 0.5 are highlighted in purple and annotated (F) Venn diagram showing overlap of SARS-CoV-2 nsp15 interactors identified in previous studies[6,9,15–18] and SARS-CoV-2 nsp15 interactors identified in this study.

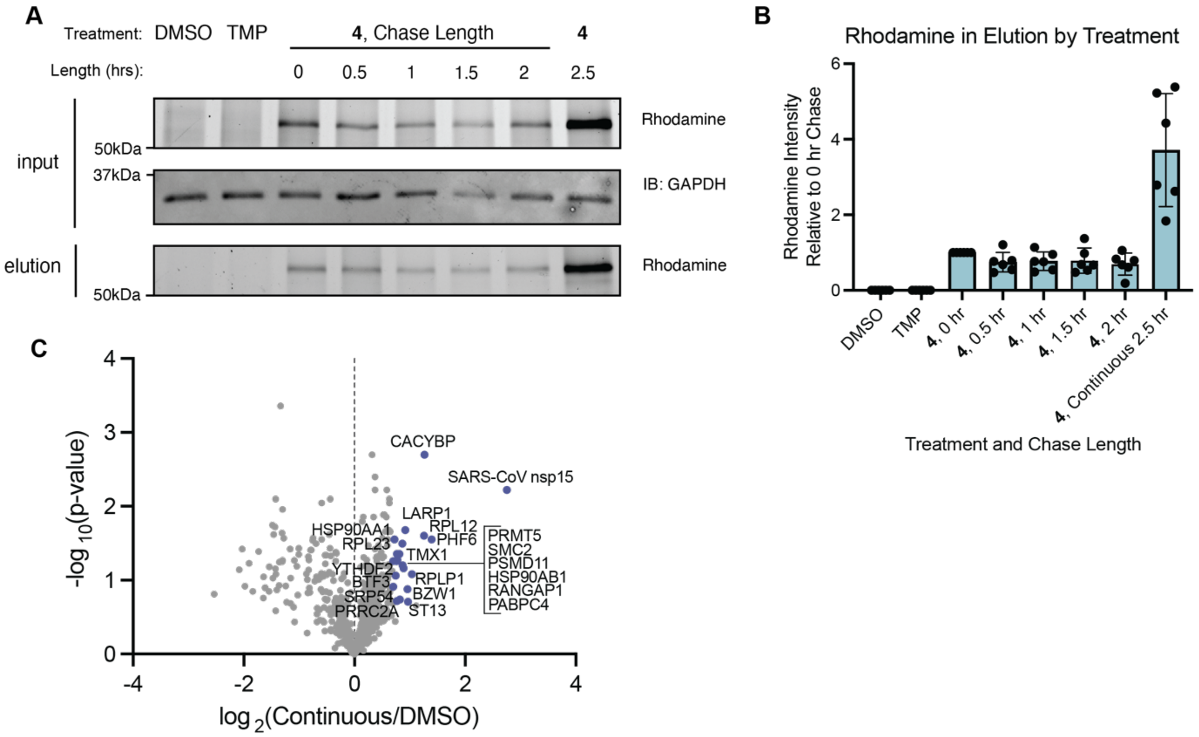

Figure S6. Mass spectrometry characterization of SARS-CoV nsp15 TRAPPED: (A) Images of rhodamine and GAPDH intensity in Western blots from FT-DHFR(DD)^{L28C}-SARS-CoV-nsp15 transfected HEK293T cells treated with DMSO, 10 μM TMP, or **4** for a 30-minute pulse. Subsequently, samples were chased for the duration indicated with excess TMP. Lysates were reacted with TAMRA-Azide-PEG-Desthiobiotin and enriched via streptavidin affinity purification. Samples before (input) and after affinity purification (elution) were resolved by SDS-PAGE and Western blots. The right-most lane shows the positive control sample treated continuously with **4** for 2.5 hours. DMSO and TMP treated samples are shown as negative controls. (B) Quantification of rhodamine intensity in the elution SDS-Page gel from (A) relative to 0-hour chase sample for 6 replicates of TRAPPED. (C) Volcano plot comparing the enrichment of nsp15 interactors in continuous sample to vehicle control. Interactors with $\log_2$(fold change) greater than 0.7 and $-\log_{10}$(p-value) greater than 0.7 are highlighted in purple and annotated.

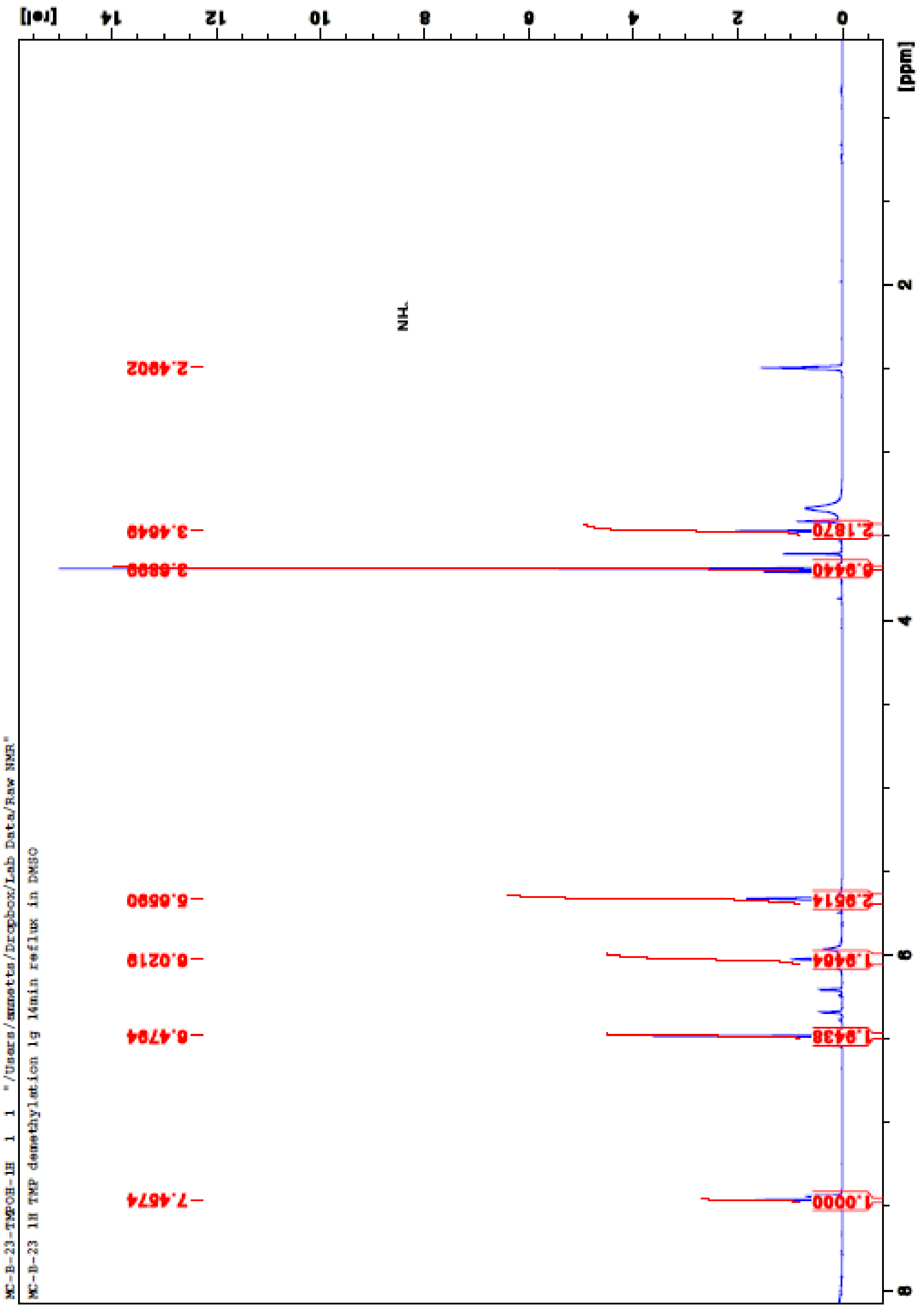

Figure S7. ^{1}H NMR of Compound **1** in DMSO-d6

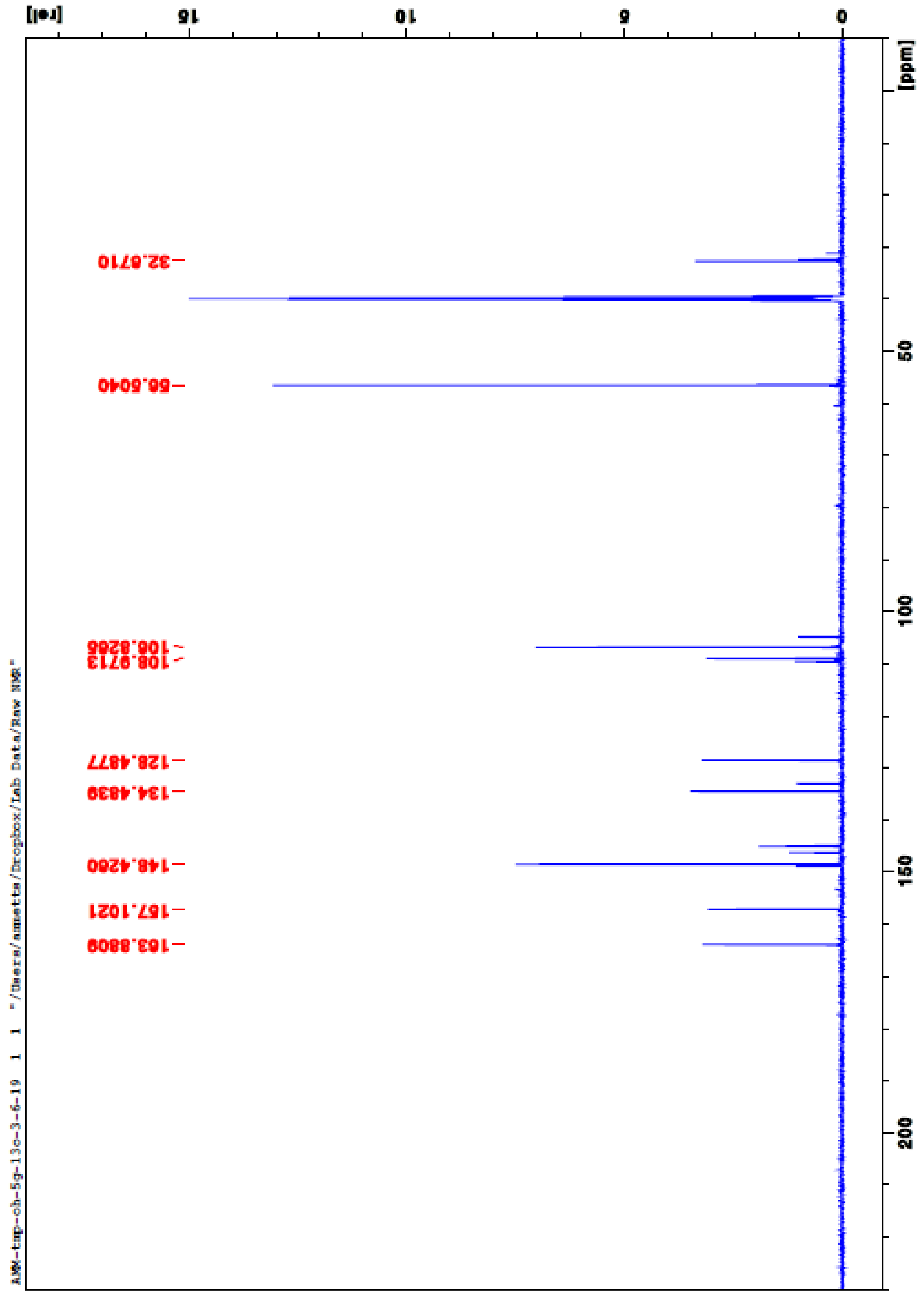

Figure S8. ^{13}C NMR of Compound **1** in DMSO-d6

Peak #	Time	Area % UV215	Area % UV254	Area % TIC(+)	BPM	Area Abs UV215	Area Abs UV254	Area Abs TIC(+)
1	0.098	90.5	100.0	100.0	277.3	2705.60	925.71	685692.81
2	0.137	9.5	0.0	0.0	157.2	285.32	0	0

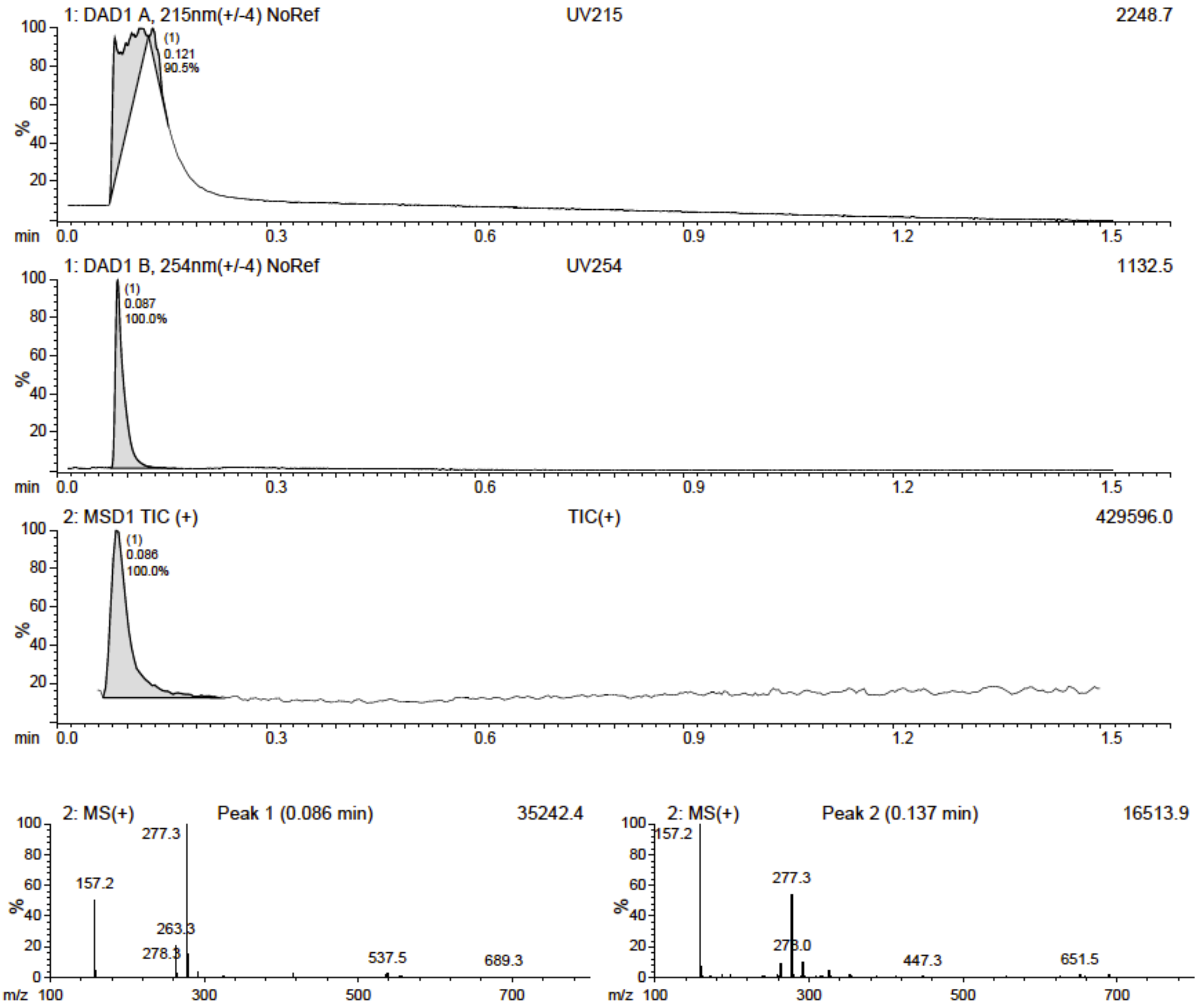

Figure S9. LC/MS of Compound **1** (expected MW: 277.1)

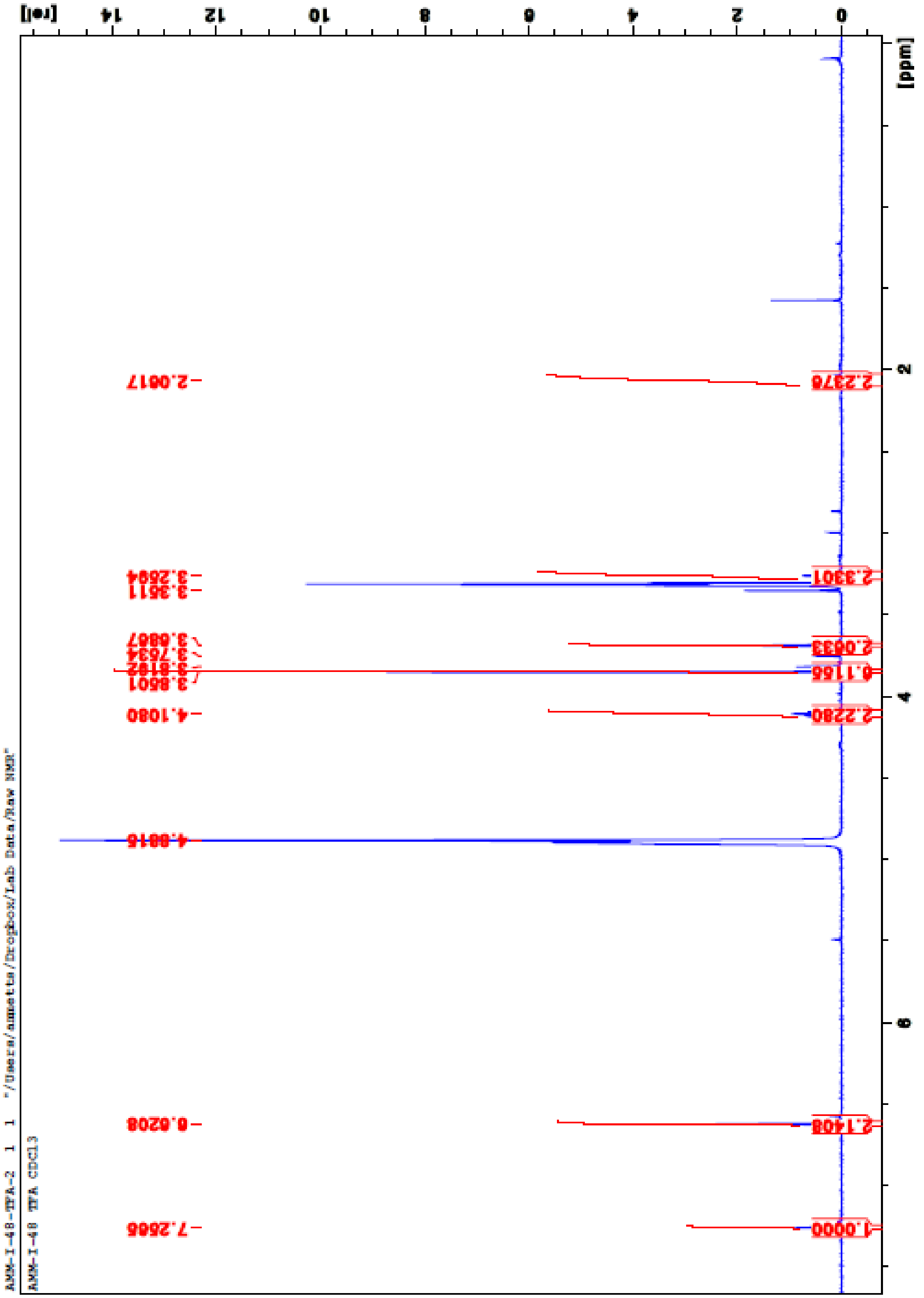

Figure S10. ^{1}H NMR of Compound **2** in MeOD

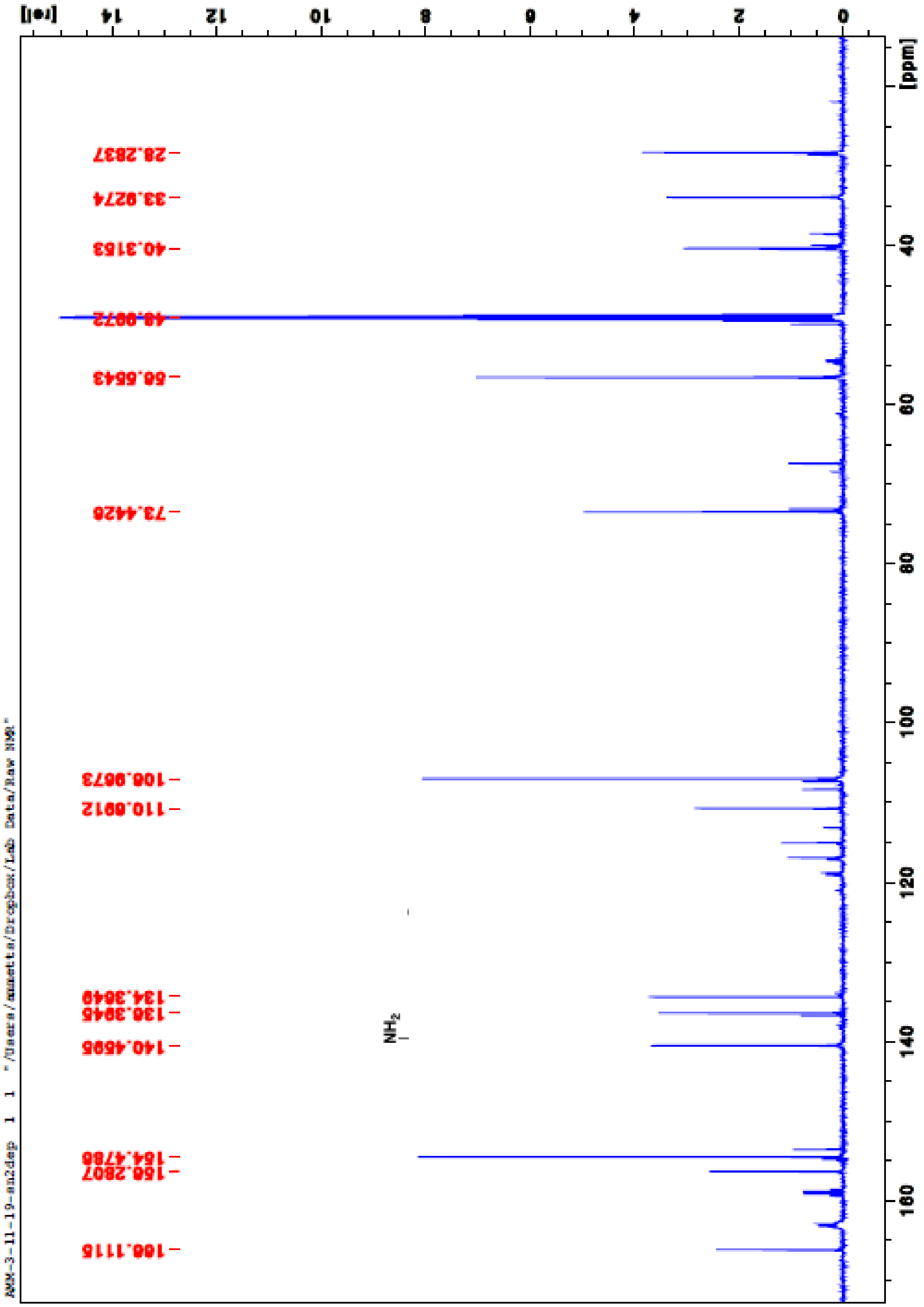

Figure S11. ^{13}C NMR of Compound **2** in MeOD

Peak #	Time	Area % UV215	Area % UV254	Area % TIC(+)	BPM	Area Abs UV215	Area Abs UV254	Area Abs TIC(+)
1	0.085	65.8	100.0	74.6	334.3	190.13	19.78	167996.08
2	0.291	34.2	0.0	0.0	214.3	98.97	0	0
3	1.066	0.0	0.0	16.2	215.3	0	0	36519.75
4	1.454	0.0	0.0	9.2	282.4	0	0	20617.54

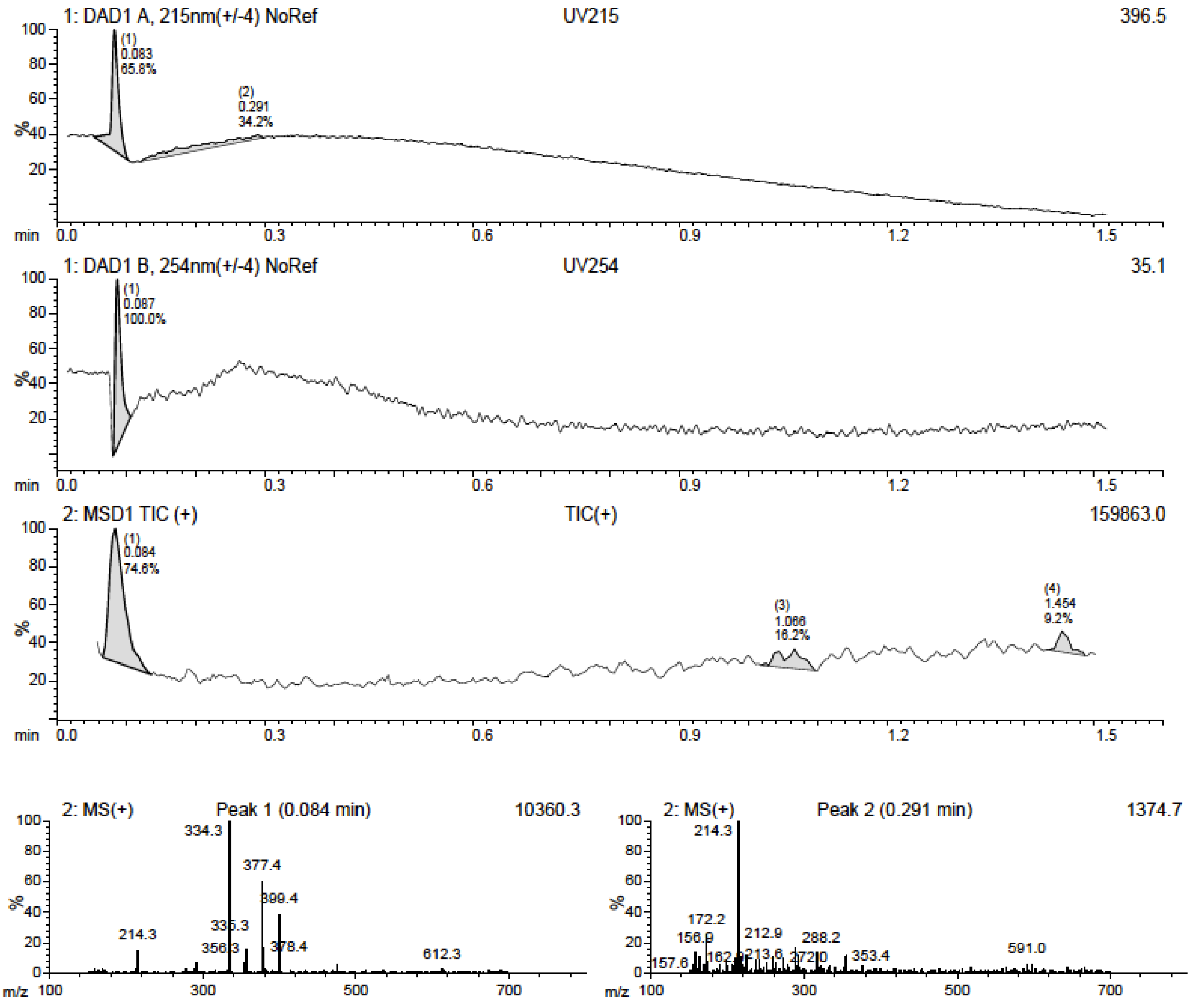

Figure S12. LC/MS of Compound **2** (expected MW: 334.2)

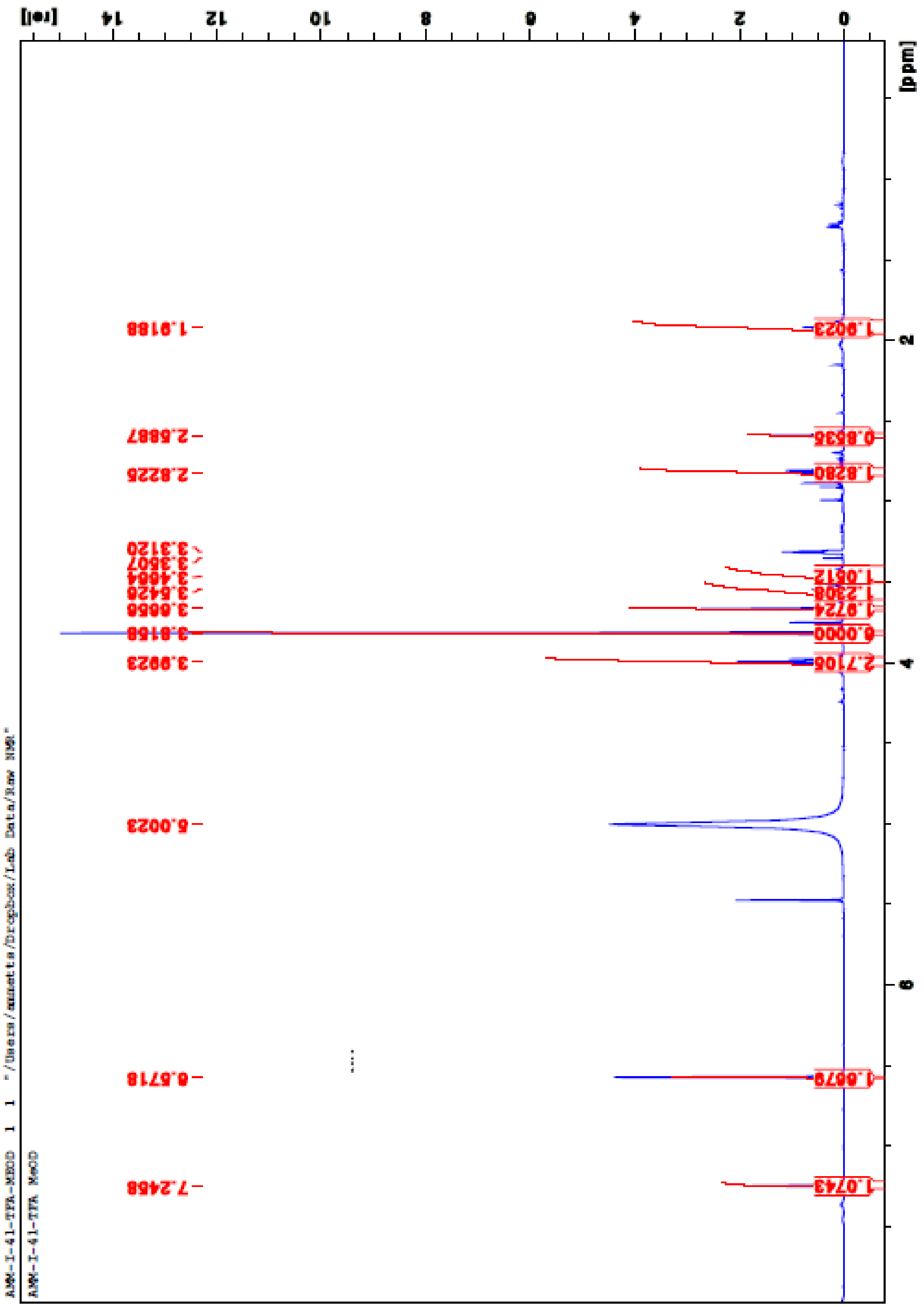

Figure S13. ^{1}H NMR of Compound **3** in MeOD

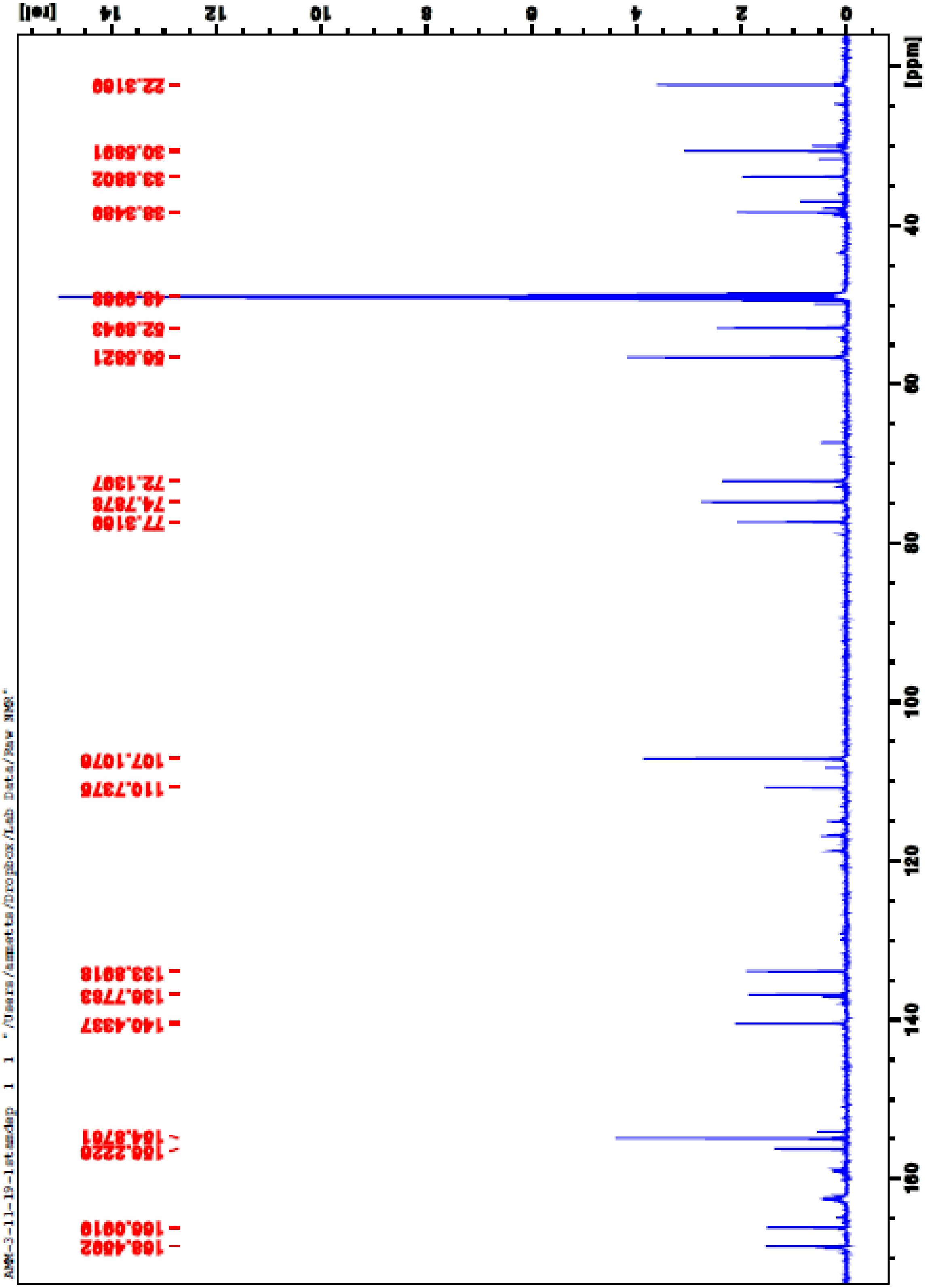

Figure S14. ^{13}C NMR of Compound **3** in MeOD

Peak #	Time	Area % UV215	Area % UV254	Area % TIC(+)	BPM	Area Abs UV215	Area Abs UV254	Area Abs TIC(+)
1	0.081	11.6	0.0	6.8	215.3	32.39	0	92612.98
2	0.120	9.4	0.0	0.0	215.4	26.18	0	0
3	0.242	6.4	0.0	6.4	214.3	17.96	0	87812.61
4	0.833	63.8	100.0	46.2	429.3	177.80	26.08	629264.50
5	0.993	4.7	0.0	11.2	568.2	13.02	0	153004.38
6	1.061	4.1	0.0	9.3	568.4	11.46	0	125998.68
7	1.393	0.0	0.0	20.1	353.4	0	0	273372.22

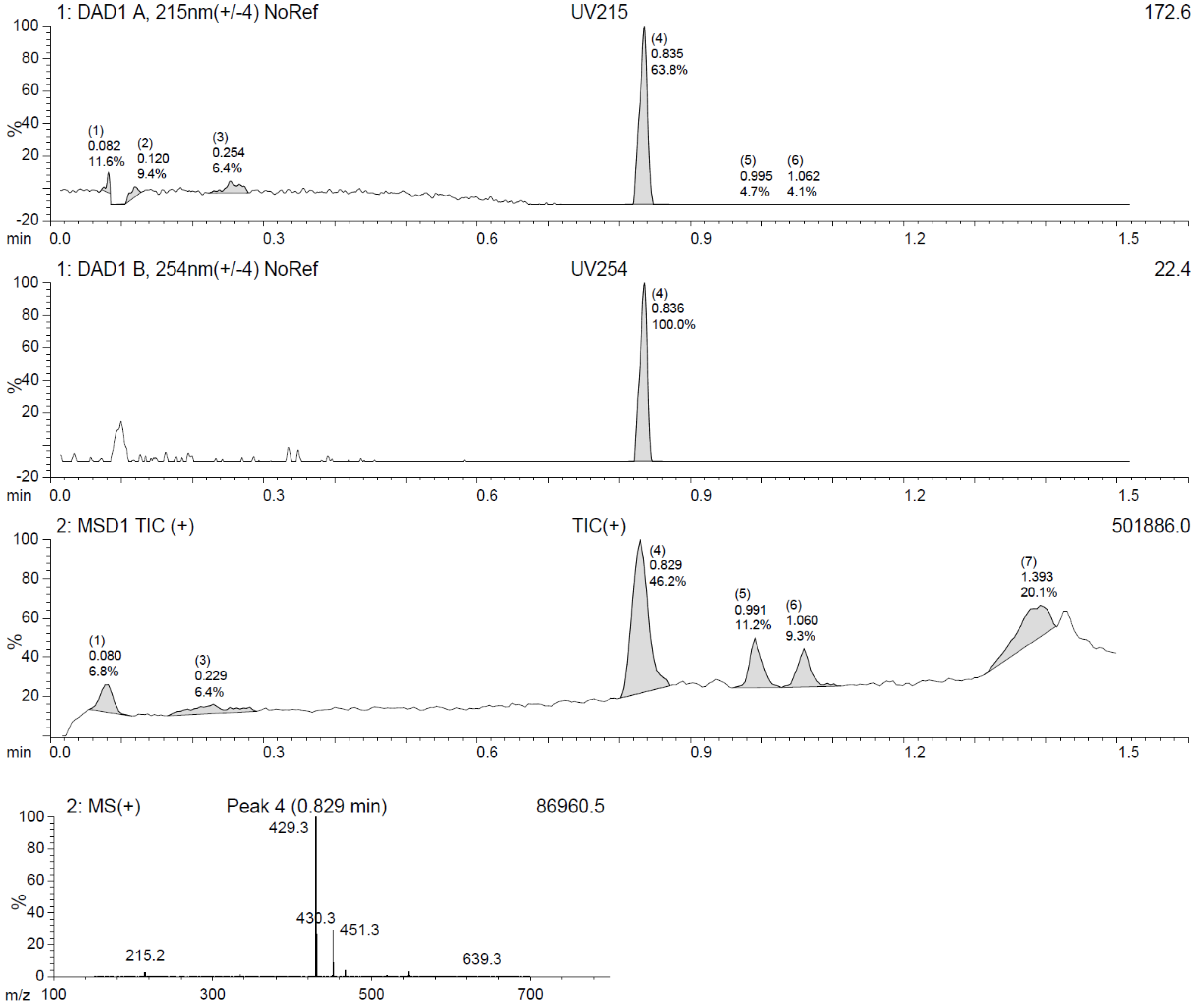

Figure S15. LC/MS of Compound **3** (expected MW: 429.2)

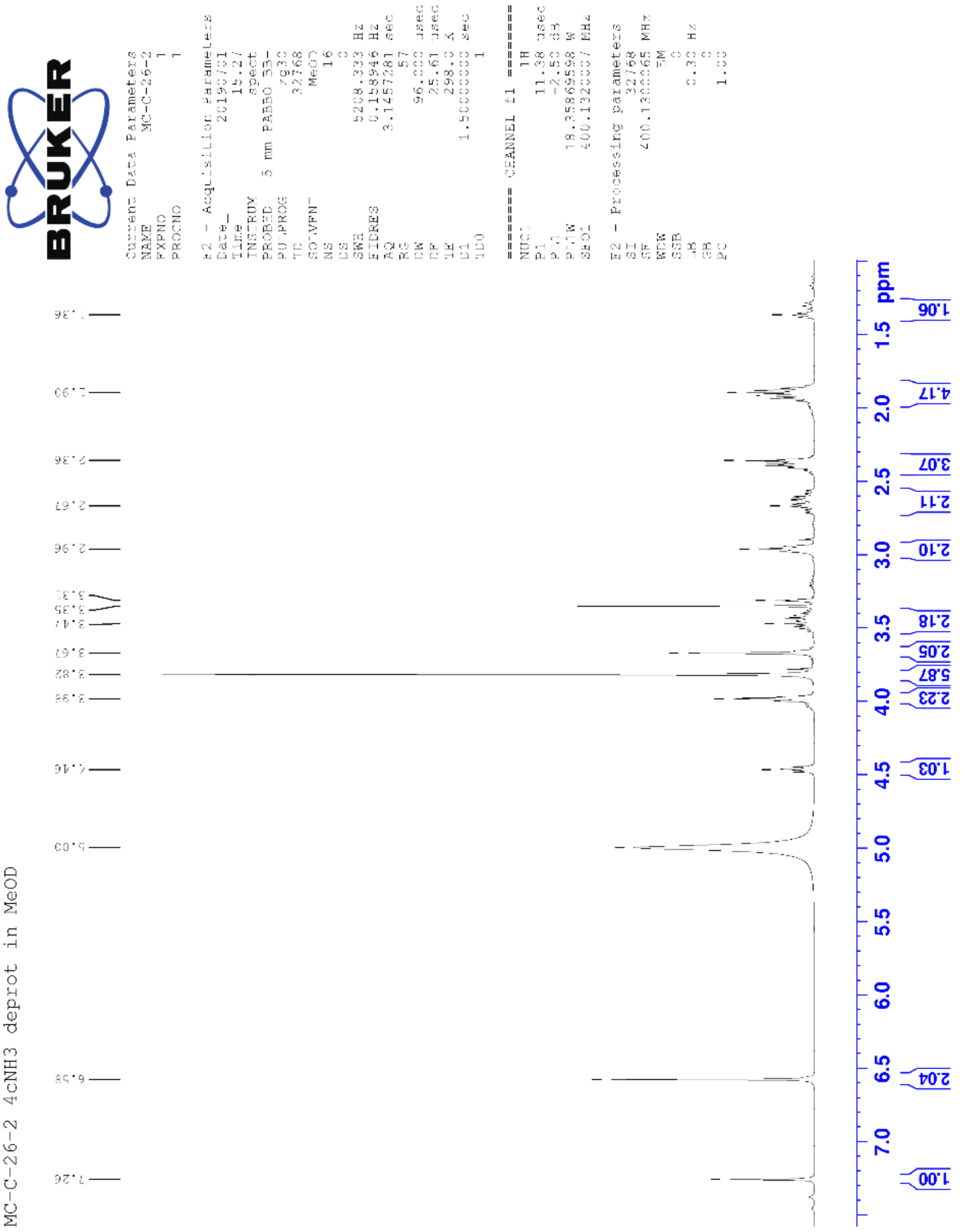

Figure S16. ^{1}H NMR of Compound **6a** in MeOD

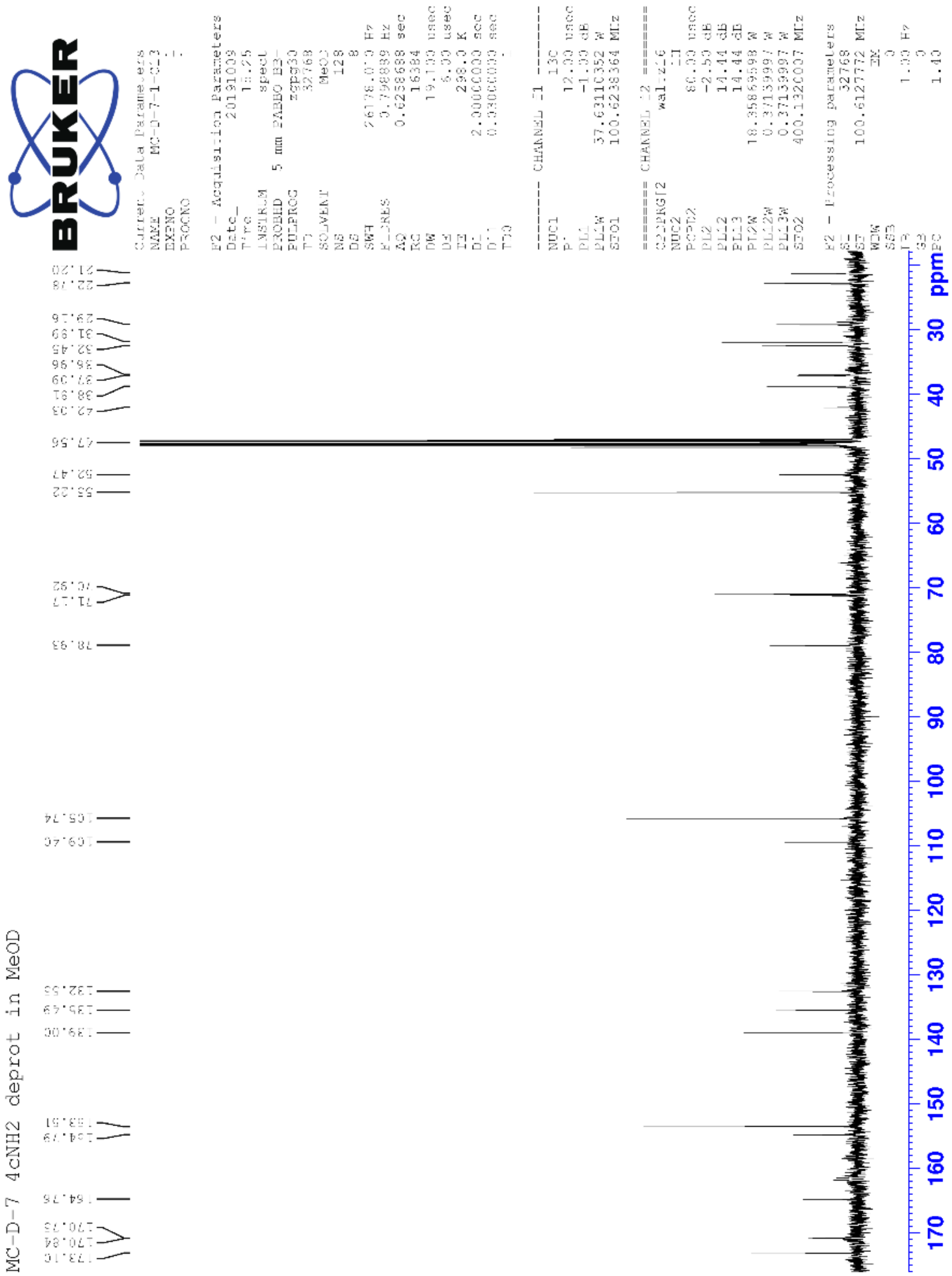

Figure S17. ^{13}C NMR of Compound **3a** in MeOD

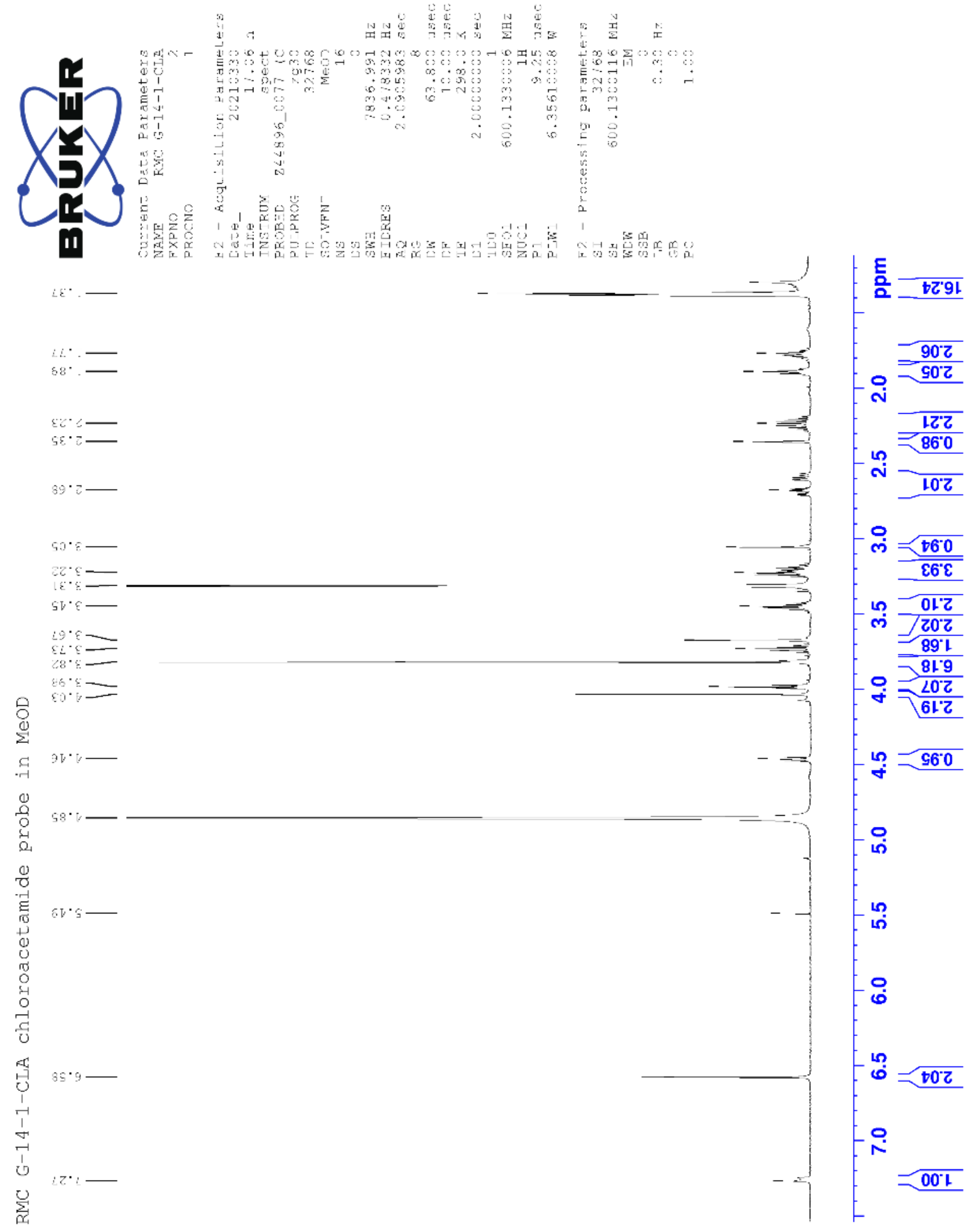

Figure S18. ^{1}H NMR of Compound **4** in MeOD

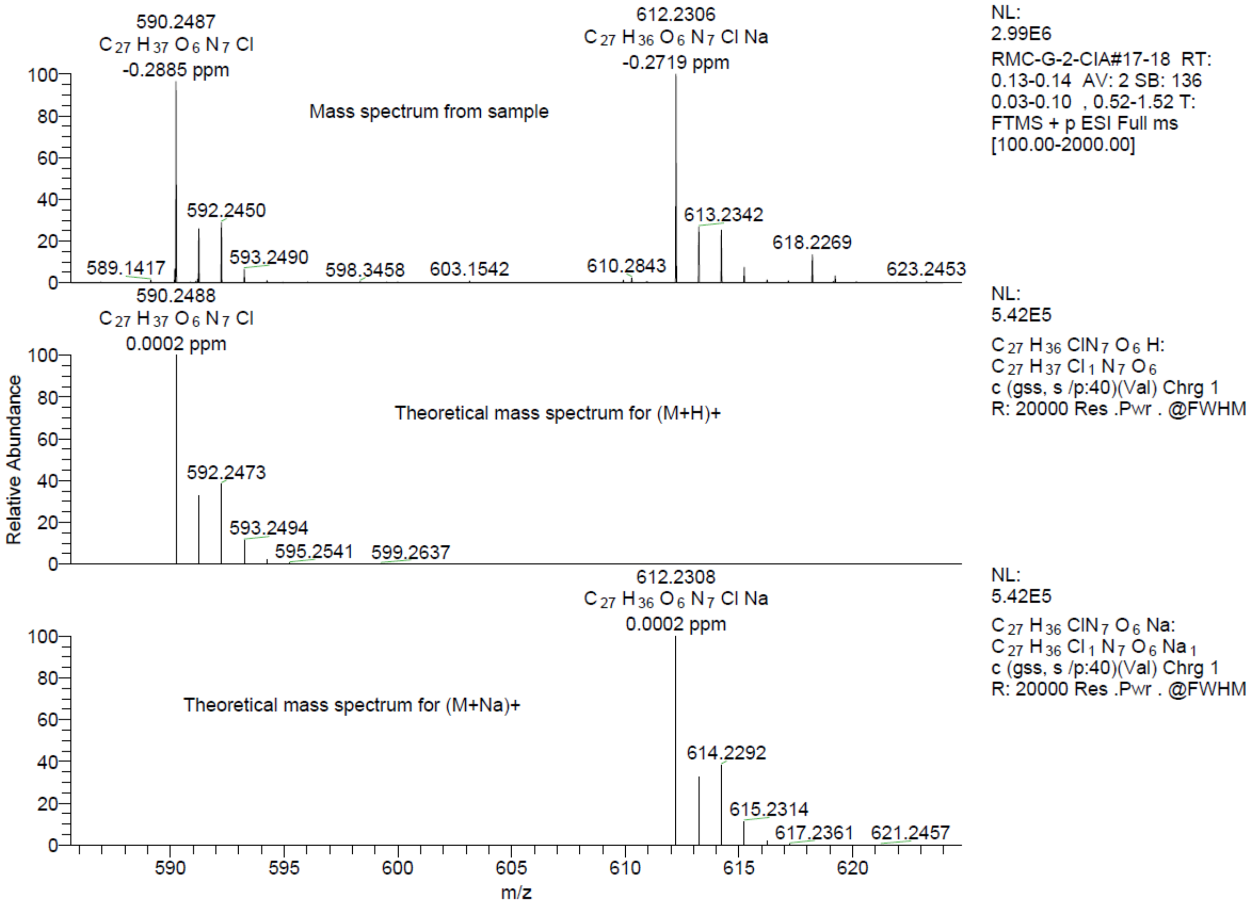

Figure S19. HRMS of Compound **4** (expected spectra shown)

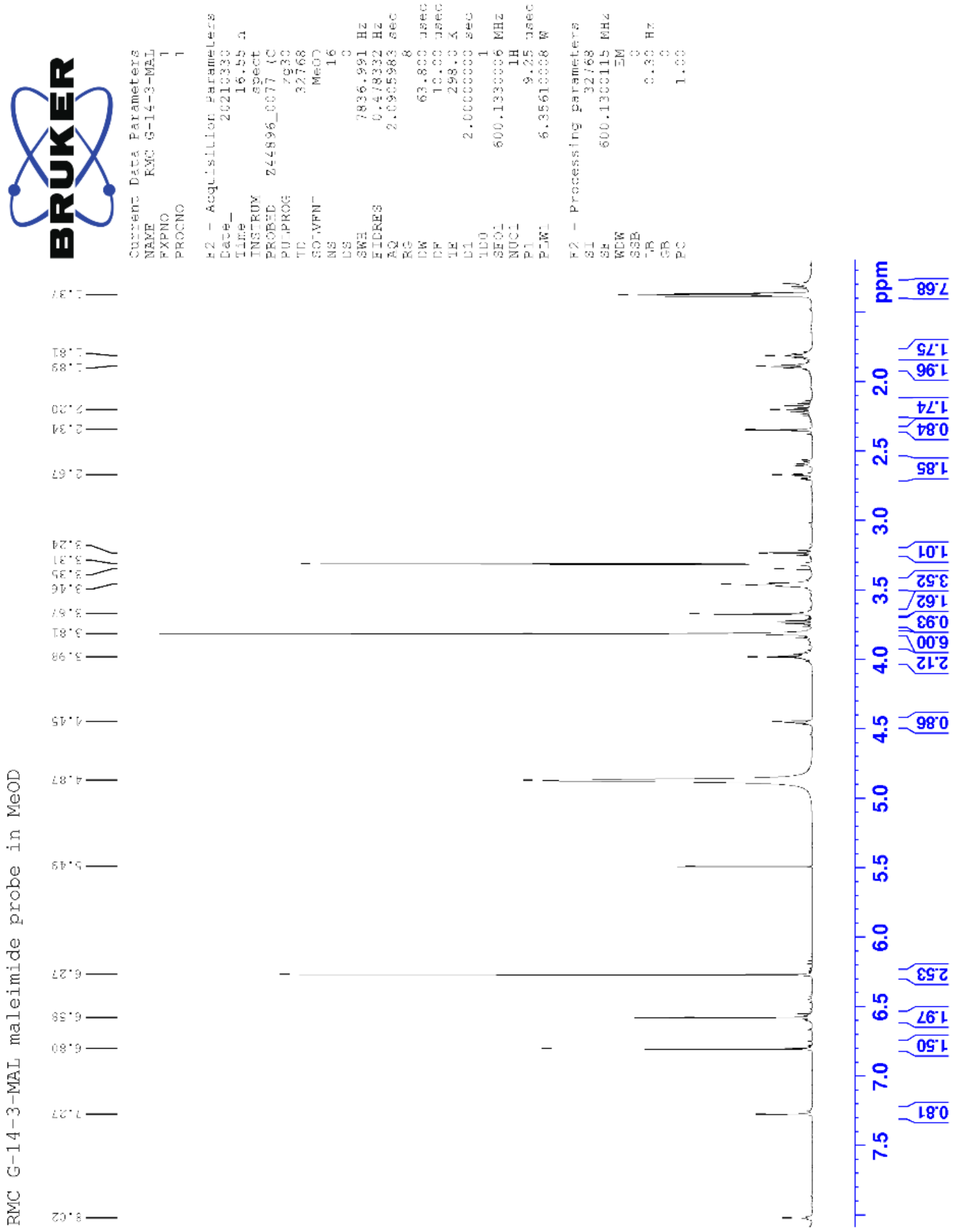

Figure S20. ^{1}H NMR of Compound **5** in MeOD

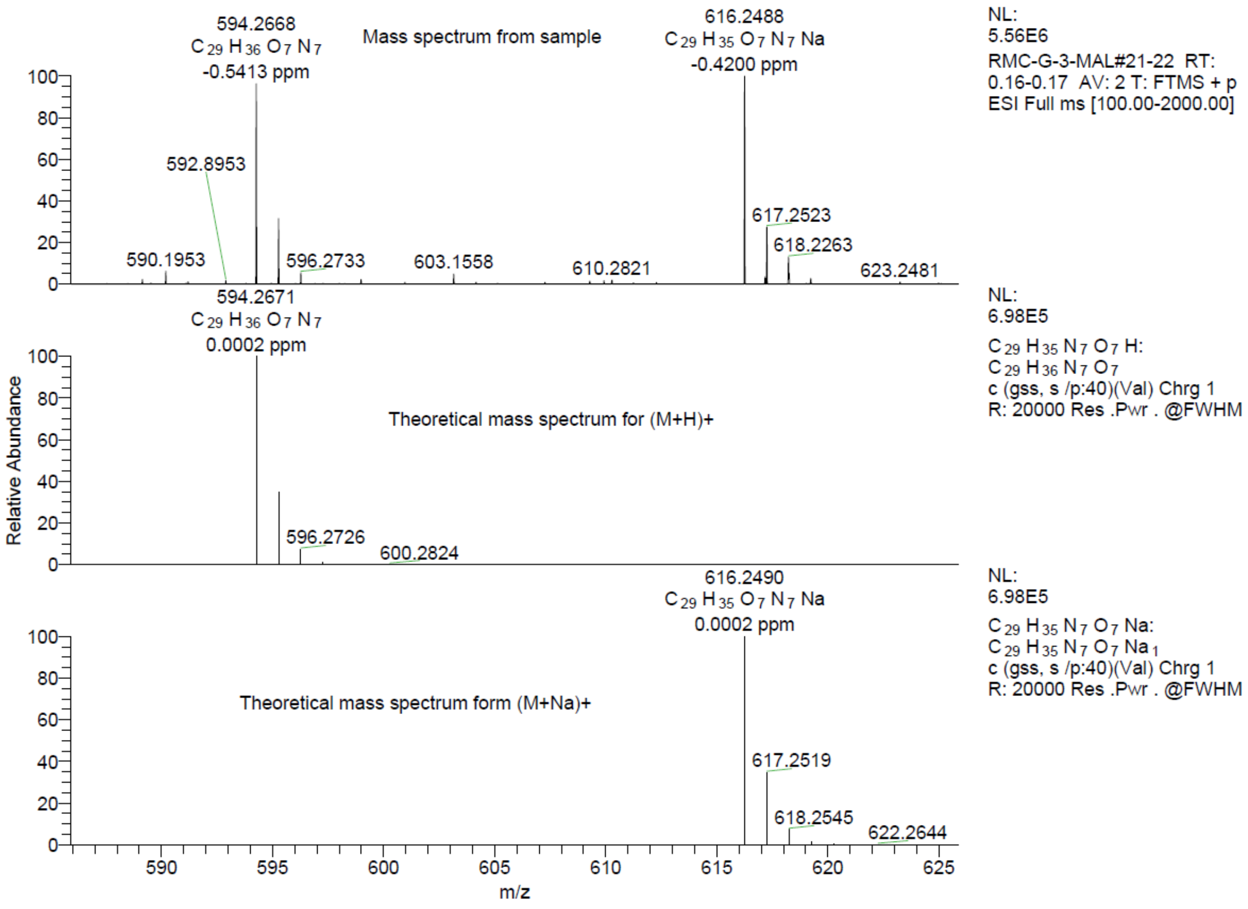

Figure S21. HRMS of Compound **5** (expected spectra shown)

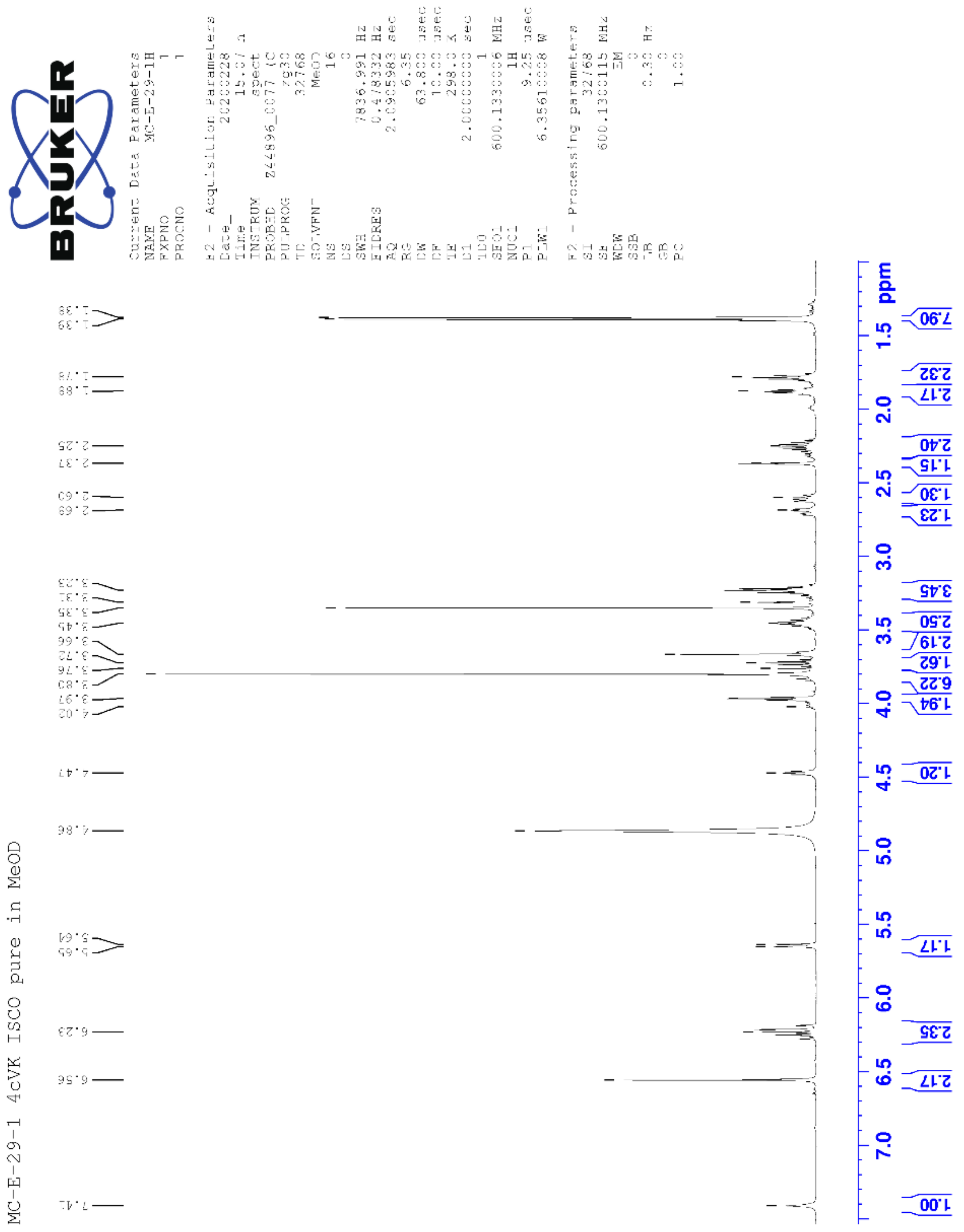

Figure S22. ^{1}H NMR of Compound **6** in MeOD

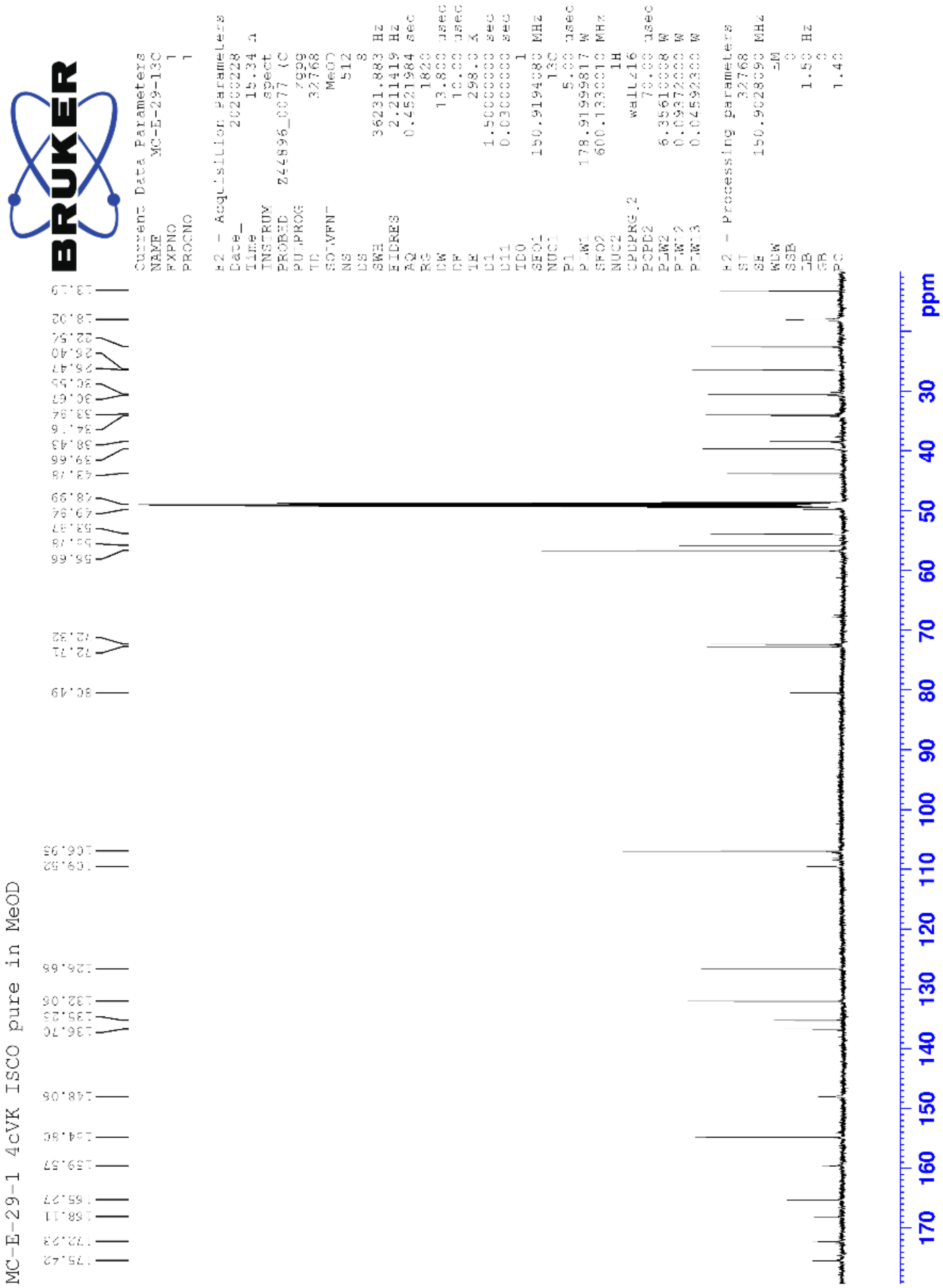

Figure S23. ^{13}C NMR of Compound **6** in MeOD

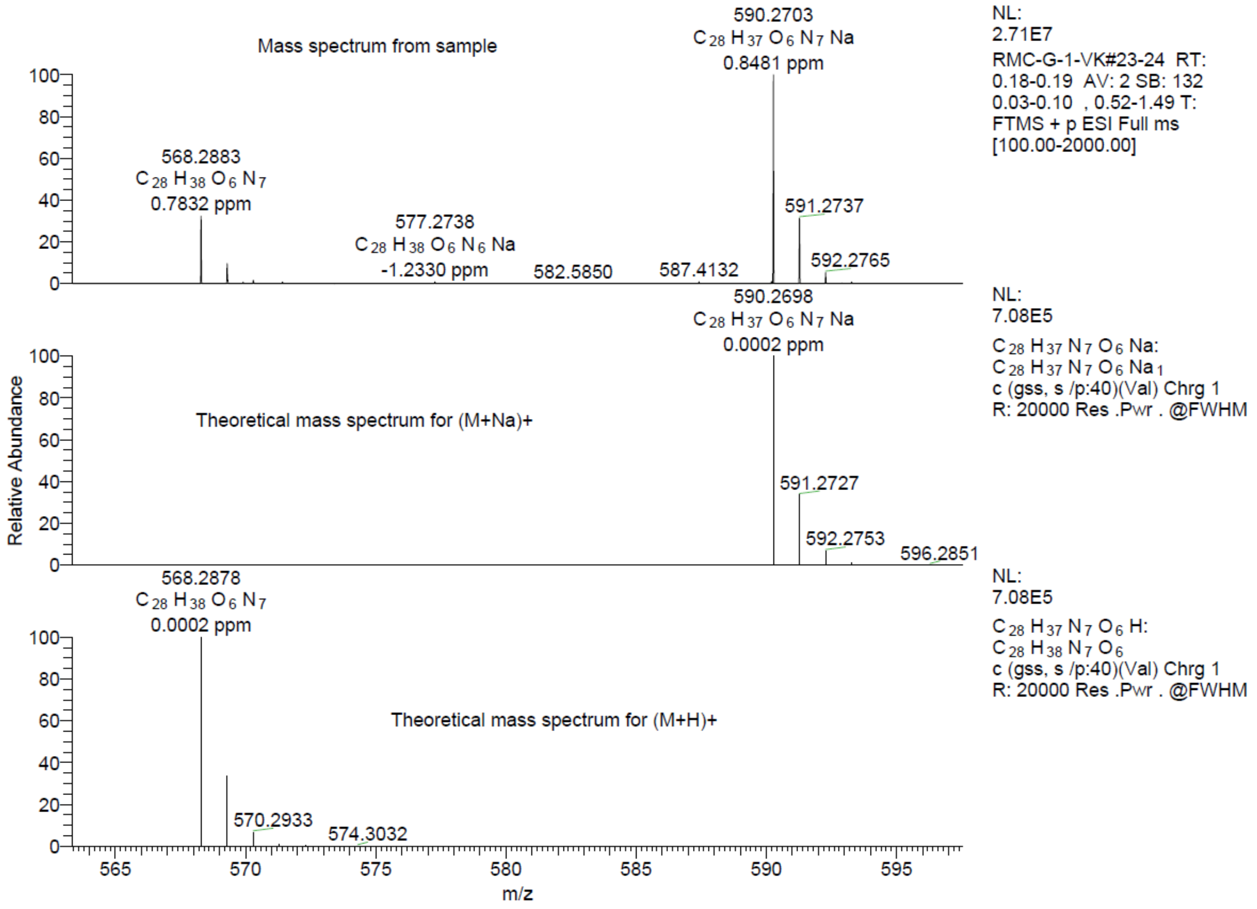

Figure S24. HRMS of Compound **6** (expected spectra shown)
